# Integrated proteogenomic characterization across major histological types of pituitary neuroendocrine tumors

**DOI:** 10.1038/s41422-022-00736-5

**Published:** 2022-10-28

**Authors:** Fan Zhang, Qilin Zhang, Jiajun Zhu, Boyuan Yao, Chi Ma, Nidan Qiao, Shiman He, Zhao Ye, Yunzhi Wang, Rui Han, Jinwen Feng, Yongfei Wang, Zhaoyu Qin, Zengyi Ma, Kai Li, Yichao Zhang, Sha Tian, Zhengyuan Chen, Subei Tan, Yue Wu, Peng Ran, Ye Wang, Chen Ding, Yao Zhao

**Affiliations:** 1 State Key Laboratory of Genetic Engineering and Collaborative Innovation Center for Genetics and Development, School of Life Sciences, Institute of Biomedical Sciences, Human Phenome Institute, Zhongshan Hospital, Fudan University, Shanghai, China; 2 Department of Neurosurgery, Huashan Hospital, Shanghai Medical College, Fudan University, Shanghai, China; 3 National Center for Neurological Disorders, Huashan Hospital, Shanghai Medical College, Fudan University, Shanghai, China; 4 State Key Laboratory of Cell Differentiation and Regulation, Henan International Joint Laboratory of Pulmonary Fibrosis, Henan center for outstanding overseas scientists of pulmonary fibrosis, College of Life Science, Institute of Biomedical Science, Henan Normal University, Xinxiang, Henan China; 5 Department of Radiology, Huashan Hospital, Shanghai Medical College, Fudan University, Shanghai, China; 6 State Key Laboratory of Medical Neurobiology and MOE Frontiers Center for Brain Science, Institutes of Brain Science, Fudan University, Shanghai, China; 7 Shanghai Key laboratory of Brain Function Restoration and Neural Regeneration, Shanghai, China; 8 Neurosurgical Institute of Fudan University, Shanghai, China; 9 National Clinical Research Center for Aging and Medicine, Huashan Hospital, Fudan University, Shanghai, China

**Keywords:** Pituitary tumours, Proteomic analysis

## Abstract

Pituitary neuroendocrine tumor (PitNET) is one of the most common intracranial tumors. Due to its extensive tumor heterogeneity and the lack of high-quality tissues for biomarker discovery, the causative molecular mechanisms are far from being fully defined. Therefore, more studies are needed to improve the current clinicopathological classification system, and advanced treatment strategies such as targeted therapy and immunotherapy are yet to be explored. Here, we performed the largest integrative genomics, transcriptomics, proteomics, and phosphoproteomics analysis reported to date for a cohort of 200 PitNET patients. Genomics data indicate that *GNAS* copy number gain can serve as a reliable diagnostic marker for hyperproliferation of the PIT1 lineage. Proteomics-based classification of PitNETs identified 7 clusters, among which, tumors overexpressing epithelial-mesenchymal transition (EMT) markers clustered into a more invasive subgroup. Further analysis identified potential therapeutic targets, including CDK6, TWIST1, EGFR, and VEGFR2, for different clusters. Immune subtyping to explore the potential for application of immunotherapy in PitNET identified an association between alterations in the JAK1-STAT1-PDL1 axis and immune exhaustion, and between changes in the JAK3-STAT6-FOS/JUN axis and immune infiltration. These identified molecular markers and alternations in various clusters/subtypes were further confirmed in an independent cohort of 750 PitNET patients. This proteogenomic analysis across traditional histological boundaries improves our current understanding of PitNET pathophysiology and suggests novel therapeutic targets and strategies.

## Introduction

Pituitary neuroendocrine tumor (PitNET, also known as pituitary adenoma) is one of the most common intracranial tumors, with an incidence of approximately 70‒90 cases per 100,000 people.^1,2^ PitNET shows a series of clinical manifestations driven by excessive hormonal secretion and invasion of surrounding structures (e.g., II~V cranial nerves, hypothalamus, and internal carotid).^3^ Although mostly considered benign, over 40% of PitNETs are invasive at the time of surgery. It is challenging to treat PitNETs due to the difficulty of complete surgical resection and the limited availability of chemotherapy and radiotherapy options.^4^

The 2017 classification by the World Health Organization (WHO)^5^ highlighted three main differentiating transcription factors (TFs), including POU1F1 (also known as PIT1) for differentiation of somatotrophs (GH), lactotrophs (PRL) and thyrotrophs (TSH); TBX19 (also known as TPIT) for differentiation of corticotrophs (ACTH); and NR5A1 (also known as SF1) for differentiation of gonadotrophs (GN). In addition, clinically silent adenomas, which do not show hormone hypersecretion and are considered non-functioning, may also express one of three specific TFs, including silent PIT1, silent TPIT, and silent SF1.^5,6^ Null-cell adenomas (NULL) are also clinically silent, although the TFs remain unknown. Plurihormonal PitNETs produce two or more hormones, and thus cannot be well defined by cell lineages.^7,8^ The present classification of PitNET is summarized in Supplementary information, Fig. S1a. This classification also indicates several specific tumor variants that have a higher rate of recurrence, including sparsely granulated somatotroph adenoma, lactotroph adenoma in men, silent corticotroph adenoma, crooke cell adenoma, and plurihormonal PIT1 positive adenoma.^5,9^

Medical treatment options for PitNETs are limited. At present, surgery represents the first-line treatment for PitNETs, while pharmacological interventions are available for two specific PitNET subtypes, i.e., dopamine agonists for PRL PitNETs, somatostatin analogs for GH PitNETs.^2,10^ However, overall response rates to both medications are moderate, and no other agents have shown significant effects against other PitNET subtypes. Although several promising molecular targets have been identified, such as EGFR for ACTH PitNETs,^11^ more druggable targets are needed for developing effective therapies.

Genetic studies have disclosed several variants involved in tumorigenesis, such as *GNAS*, *MEN1*, *NR3C1* and *AIP*.^12^ Since 2015, our group has identified other causative mutations of PitNET in a growing list of genes that include *USP8*, *KIF5A*, *GRB10* and *CDH23*.^13–15^ Among these, mutations in *GNAS* and *USP8* are the major causative factors (i.e., present in 40%‒60%) of GH and ACTH PitNETs, respectively.^13,16,17^ However, the biological mechanism connecting copy number alterations (CNAs) and tumorigenesis remains unclear. Only three TFs (PIT1, TPIT and SF1) are currently used to delineate the major PitNET cell lineages, although patients exhibit diverse hormone secretion profiles and varying clinical prognosis, which together suggest that additional TFs may participate in tumorigenesis. While the large majority of published studies are genomic or transcriptomic analyses, an integrated multi-omics analysis can provide the comprehensive perspective necessary to identify robust pathogenesis, prognostic, and therapeutic markers for different PitNET lineages.^18,19^

Advances in integrative multi-omics strategies, such as those encompassing proteomics and phosphoproteomics profiling, in conjunction with genomic analysis, have driven therapeutic development for several different tumor types.^20–22^ In this regard, comprehensive characterization of the proteogenomic landscape is essential for progress in developing therapeutic strategies. To this end, we analyzed genomics, transcriptomics, proteomics, and phosphoproteomics datasets from 200 PitNETs and 7 anterior pituitary glands (APGs) as controls. We established a novel, molecularly unbiased classification of PitNET subtypes to understand their pathophysiological mechanisms and explore potential actionable targets for each subtype. Moreover, the data generated for these analyses will serve as an essential resource for further biological and functional investigation, as well as drug discovery for PitNET.

## Results

### Proteogenomic analyses of PitNET specimens

**Fig. 1:**
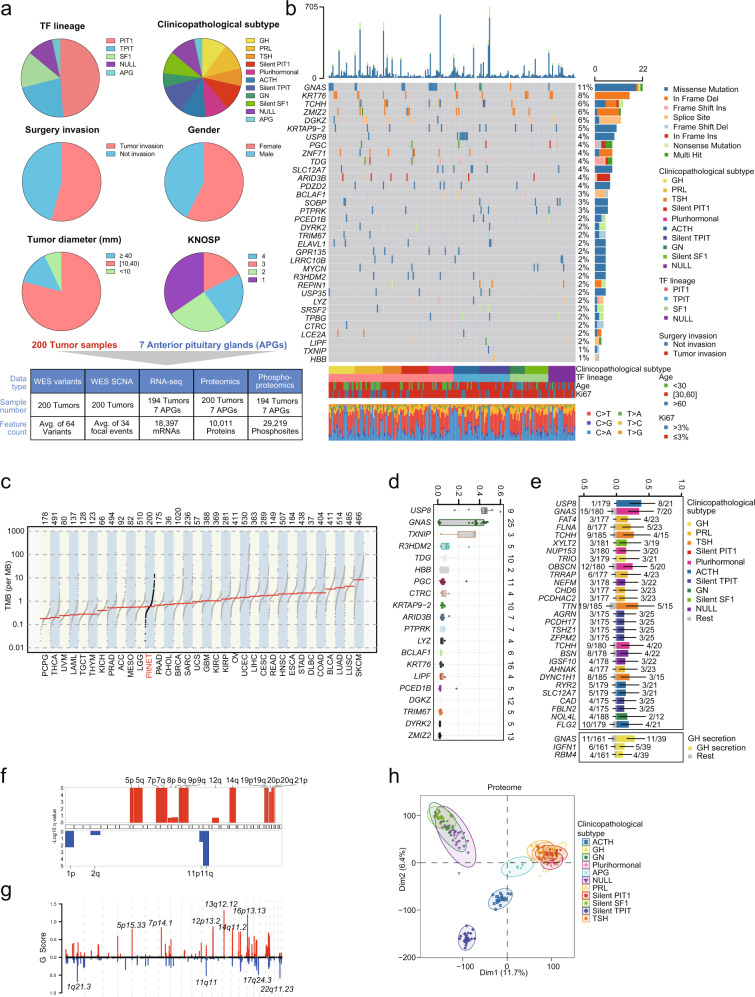
Proteogenomic landscape of PitNETs. **a** Top panel, pie charts of clinical indicators. Bottom panel, sample numbers and multi-omics datasets of the cohort. **b** Genomic profile and associated clinical features of patients with PitNETs. SMGs in this dataset identified by MutSigCV and OncodriveCLUST (q value < 0.1) are shown. Right panel, percentage of samples affected. Top panel, number of mutations per sample. Middle panel, distribution of significant mutations across sequenced samples, color coded by mutation type. Bottom panel, percentage of somatic base changes per sample. **c** Comparison of the TMB of our PitNETs cohort and 33 cancer types in TCGA studies. **d** Boxplot showing the VAF of the top 20 SMGs. **e** Bar plot showing the genes with significantly different mutation frequencies based on Fisher’s exact test by clinicopathological subtype (Fisher’s exact test, *P* value < 0.01). The numbers listed on the right side of the barplot represented the mutation frequencies in the indicated clinicopathological subtype tumors. The numbers listed on the left side of the barplot represented the mutation frequencies in the rest tumors. **f**, **g** Arm-level and focal-level amplifications and deletions. GISTIC analysis was performed to determine significant regions and genes included in the recurrent CNAs identified in patients with PitNETs. **h** PCA analysis of proteomics data from 200 PitNETs and 7 APGs based on clinicopathological subtypes.

To obtain the proteogenomic landscape of PitNET, whole-exome sequencing (WES), transcriptomics, proteomics, and phosphoproteomics datasets were collected from 200 fresh-frozen tumors and 7 APGs as controls, based on pathological criteria (see Materials and methods). Clinicopathological features, including TF lineage, clinicopathological subtypes, surgery invasion status, patient gender, tumor diameter, and KNOSP grade are summarized in Supplementary information, Table S1. Figure 1a illustrates the sample distribution across the three TF lineages and NULL, which were further divided into 10 clinicopathological subtypes: PIT1 lineage (*n* = 101, including 21 GH, 23 PRL, 15 TSH, 22 silent PIT1, and 20 plurihormonal), TPIT lineage (*n* = 46, including 21 ACTH, and 25 silent TPIT), SF1 (*n* = 31, including 12 GN and 19 silent SF1), and NULL (*n* = 22) (Supplementary information, Fig. S1b).

WES was conducted on 200 tumor tissues and paired peripheral blood mononuclear cells (PBMCs) to identify possible genetic variants in the cancer genome. RNA sequencing (RNA-seq) was carried out for 194 tumors and 7 APGs. A mass spectrometry (MS)-based label-free quantitative (LFQ) method was used to characterize the proteomes of the 200 tumors and 7 APGs. A Fe-NTA-enrichment-based strategy was employed for phosphoproteomics profiling of 194 tumors and 7 APGs (Fig. 1a; Supplementary information, Fig. S1b).

### Overview of the proteogenomic landscape of PitNET

WES data analysis revealed 7333 mutated genes, including 11,092 non-silent point mutations and 419 small insertions or deletions (indels) (Fig. 1b; Supplementary information, Table S1). In the 200 patients, we observed several significantly mutated genes (SMGs, q < 0.1) associated with PitNET functions, including *GNAS* (11%), *KRT76* (8%), *TCHH* (6%), *ZMIZ2* (6%), *DGKZ* (6%), *KRTAP9-2* (5%), and *USP8* (4%) (Fig. 1b). Examination of the proportion of somatic base changes revealed that PitNET patients carried a high proportion of C > A transitions compared to the other five substitution types (Fig. 1b). A comparison with previous studies using The Cancer Genome Atlas (TCGA)^23^ indicated that, in this study, the tumor mutation burden (TMB) in the PitNET cohort remained a lower-middle level among the 33 cancer types (Fig. 1c).

We next compared the variant allele frequencies (VAFs) of the SMGs and found that *USP8* mutation (median: 0.45) ranked first, followed by *GNAS* mutation (median: 0.36) (Fig. 1d). All nucleotide variants in these two genes detected in PitNET patients were previously reported.^15,19^ Enrichment analysis using Fisher’s exact test to identify mutations associated with clinicopathological subtype showed that *USP8* mutations were enriched in the ACTH subtype (*P* = 3.12e‒8), while *GNAS* mutations were enriched in the plurihormonal subtype (plurihormonal subtype vs remaining samples, *P* = 0.002) and GH subtype (GH subtype vs remaining samples, *P* = 0.00059) (Fig. 1e). Somatic CNA analysis identified arm-level amplifications (Chr 5, 7, 8, 9, 12q, 14q, 19, 20, 21p) and deletions (Chr 1p, 2q, 11) (Fig. 1f). Focal peaks included amplifications of 5p15.33, 7p14.1, 12p13.2, 13q12.12, 14q11.2 and 16p13.13 and deletions of 1q21.3, 11q11, 17q24.3 and 22q11.23, among others (see Materials and methods; Fig. 1g; Supplementary information, Table S2).

Our transcriptomics, proteomics, and phosphoproteomics datasets exhibited a unimodal distribution and passed the quality control (QC) procedure (Supplementary information, Fig. S1c). RNA-seq identified 18,397 genes with fragments per kilobase of transcript per million fragments mapped (FPKM) values over 1, providing an opportunity to explore the relationship between transcriptome and proteome. For proteomics analysis, whole-cell extracts of human embryonic kidney-derived HEK293T cells were used as controls for quality. Quantitative MS analysis of HEK293T cells confirmed the robustness and consistency of the MS data, indicated by a high Spearman’s correlation coefficient of 0.91 among the proteomes of QC samples (Supplementary information, Fig. S1d). Moreover, the dataset used in this study provided in-depth coverage of the human proteome. A total of 10,011 proteins (with ≥ 2 unique peptides per protein) were identified in the 200 tumors and 7 APGs, while a total of 29,219 phosphosites were detected, corresponding to 5483 phosphoproteins. Among them, 6160 proteins and 9905 phosphosites from 3276 phosphoproteins were selected for downstream analysis based on their presence in more than 50% of cases of at least one clinicopathological tumor subtype.

Principal component analysis (PCA) of our multi-omics data revealed a significant separation between the PIT1 lineage (GH, PRL, TSH, silent PIT1, and plurihormonal subtypes) and the APG, ACTH, silent TPIT, and other PitNETs at the proteomics level, whereas the PIT1 lineage showed higher similarity to the APG group at the transcriptomics and phosphoproteomics levels (Fig. 1h; Supplementary information, Fig. S1e, f). Transcriptomics and proteomics data further indicated that lineage-specific TFs (PIT1, TPIT, and SF1) and hormone-related genes (*GH1*, *PRL*, *TSHB*, *POMC*, *LHB*, and *FSHB*) were expressed in specific clinicopathological subtypes (Supplementary information, Fig. S1g). These cumulative results thus provide a multi-omics landscape to improve our understanding of the molecular mechanisms of PitNETs.

### Impact of genomic alterations on the transcriptome, proteome, and phosphoproteome

**Fig. 2:**
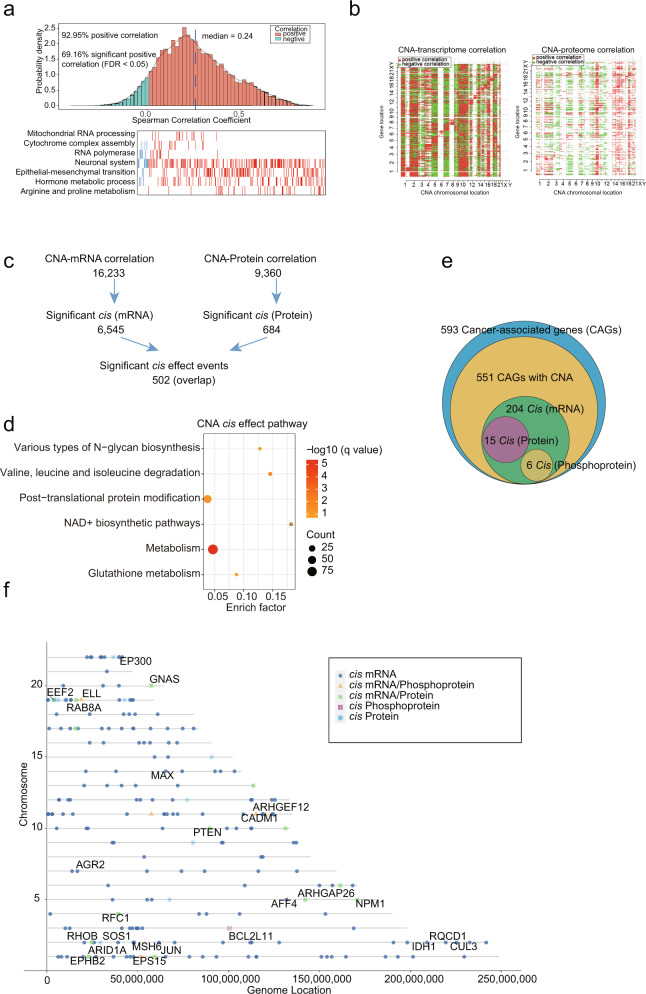
Impact of CNAs on the transcriptome, proteome and phosphoproteome of PitNETs. **a** Gene-wise mRNA-protein Spearman’s correlation in tumors. Red, pathways involving positively correlated genes; blue, pathways involving negatively correlated genes (Spearman’s correlation, FDR < 0.05). **b** The correlation of CNAs to mRNA (left) or protein abundance (right), with significant positive correlations in red and negative correlations in green (Spearman’s correlation, FDR < 0.05). Genes were sorted by chromosomal location on the *x-* and *y*-axes. **c** Cascading effects of CNAs and the overlap between *cis* events via the transcriptome and proteome analyses (Spearman’s correlation, FDR < 0.05). **d** Prioritized *cis* effect CNA drivers were used for pathway enrichment analysis in ConsensusPathDB. **e** Venn diagram showing the CAGs with significant CNA *cis* effects via multi-omics data analyses (Spearman’s correlation, FDR < 0.1). **f** Genes with cascading copy number *cis* regulation of their cognate mRNA, protein, and phosphoprotein levels. Shapes indicate the *cis* effects across the indicated datasets.

Correlation analysis of the paired transcriptomics and proteomics datasets showed that 92.95% of 6115 mRNA-protein pairs were positively correlated in tumor samples. Genes with strong correlations were enriched in several pathways related to neuronal system, epithelial-mesenchymal transition (EMT), and hormone metabolic process, which indicate that these pathways are overrepresented in PitNETs (Fig. 2a). In addition, the global mRNA-protein correlation was moderate with sample-wise median spearman correlations of 0.45 and the correlation of each clinicopathological subtypes ranged from 0.42 to 0.46 (Supplementary information, Fig. S2a, b), which were consistent with previous reports.^21,24^

We examined the regulatory effects of 23,109 somatic CNAs on mRNA, protein, and phosphoprotein abundances of genes at the same loci (*cis* effects) and genes at other loci in the genome (*trans* effects) (see Materials and methods; Fig. 2b; Supplementary information, Fig. S2c and Table S2). We observed *cis* effects for 6545 and 684 CNAs affecting mRNAs and proteins, respectively. Among them, 502 significant *cis* effect events overlapped (Spearman’s correlation, FDR < 0.05) (Fig. 2c; Supplementary information, Table S2); these 502 genes were enriched in pathways related to post-translational protein modification, NAD^+^ biosynthesis, and metabolism (Fig. 2d). We then assessed how CNA events influenced mRNA, protein and phosphoprotein abundances of cancer-associated genes (CAGs) via either *cis* or *trans* effects, focusing on alterations in 593 previously described genes (Supplementary information, Table S2).^25^ We found that CNAs have *cis* effects on both mRNA and protein abundances of 15 CAGs, while 6 CAGs showed significant overlapped CNA *cis* effects (FDR < 0.1) at the mRNA and phosphoprotein levels (Fig. 2e). Figure 2f shows the annotations of these 21 CAGs. The *cis* or *trans* effects of these 21 genetic alterations were also comprehensively investigated (Supplementary information, Fig. S2d, e). In particular, we observed that *GNAS* copy number had *cis* effects on GNAS, and *trans* effects on EEF2, ELL, and RAB8A at the mRNA and protein levels (Supplementary information, Fig. S2d).

### Impact of *GNAS* mutation and *GNAS* copy number gain in the PIT1 lineage

**Fig. 3:**
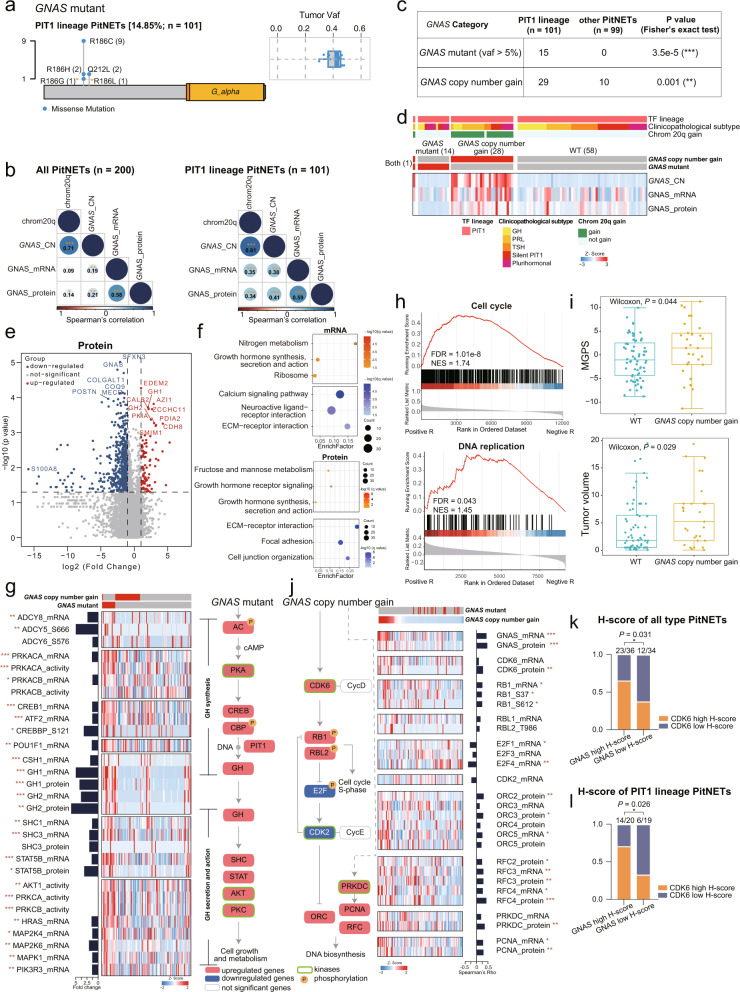
Impact of *GNAS* mutation and *GNAS* copy number gain in the PIT1 lineage. **a** Lollipop plot and boxplot showing the position and tumor VAF of the *GNAS* mutation in the PIT1 lineage. **b** Spearman’s correlation of chromosome 20q and the copy number, mRNA expression and protein abundance of *GNAS* in all PitNET samples and PIT1 lineage samples. Spearman’s correlation, *P < 0.05, **P < 0.01, ***P < 0.001. **c** Distribution of *GNAS* altered samples in different categories among the PIT1 lineage and other lineages (Fisher’s exact test, **P < 0.01, ***P < 0.001). **d** Heatmap visualizing multi-omics profiles of the levels of *GNAS* copy number, mRNA expression and protein abundance. **e** Volcano plots displaying the differentially expressed proteins in *GNAS* mutant and *GNAS* WT patients after applying a two-fold change in expression with *P* < 0.05 (Wilcoxon rank-sum test). Proteins significantly enriched in the *GNAS* mutant and *GNAS* WT patients are represented as red/blue-filled dots. **f** Pathways enriched for the differentially expressed mRNAs and proteins. Pathways that were significantly upregulated/downregulated in the *GNAS* mutants are represented as red/blue-filled dots. **g** Heatmap of multi-omics features of GH secretion-related genes. The pathway diagram on the right depicts how the features included in the heatmap regulate GH synthesis, secretion and activity. Red boxes indicate upregulated genes and blue boxes indicate downregulated genes. Green rectangles indicate kinases and orange circles indicate phosphorylated proteins. Bar chart next to the heatmap shows the fold changes of *GNAS* mutant/WT (*P < 0.05, **P < 0.01, ***P < 0.001). **h** GSEA plots for proliferation-related pathways based on the rank of *GNAS* copy number-mRNA (bottom) or protein (upper) abundance correlations. **i** Boxplots showing the difference of MGPS and tumor volume between WT and *GNAS* copy number gain group. The significance was calculated by Wilcoxon test. **j** Heatmap of multi-omics features of proliferation-related genes. The pathway diagram on the left depicts how the features included in the heatmap regulate cell cycle S-phase and DNA biosynthesis. Red boxes indicate upregulated genes and blue boxes indicate downregulated genes. Green rectangles indicate kinases and orange circles indicate phosphorylated protein. Bar chart next to the heatmap shows the Spearman’s correlation coefficient between *GNAS* copy number and proliferation-related genes (*P < 0.05, **P < 0.01, ***P < 0.001). **k**, **l** Bar plots showing the proportion of CDK6 high H-score cells between GNAS high H-score group and GNAS low H-score group in all PitNETs and PIT1 lineage PitNETs. The significance was calculated by Fisher’s exact test.

In our cohort, *GNAS*, enriched in the PIT1 lineage, was the most frequently mutated gene, harboring two mutation hotspots, R186C/G/L/H and Q212L (Figs. 1b, 3a). *GNAS* copy number gain, as a CAG with *cis* effect (Fig. 2f; Supplementary information, Fig. S2d), had particularly strong impacts on the PIT1 lineage (Spearman’s correlation in 101 PIT1 lineage vs all 200 PitNETs: R = 0.38, *P* = 0.0001, vs R = 0.19, *P* = 0.0071 at the mRNA level; R = 0.41, *P* = 2.29e‒05 vs R = 0.21, *P* = 0.0024 at the protein level) (Fig. 3b). Samples with either *GNAS* mutations (VAF > 5%) or *GNAS* copy number gain were significantly enriched in the PIT1 lineage (Fig. 3c). Thus, by integrating WES data, we could further divide the PIT1 lineage into three subgroups, including wild-type (WT), *GNAS* copy number gain, and *GNAS* mutant. Compared with the WT group, samples carrying *GNAS* mutation showed reduced GNAS protein levels (Wilcoxon rank-sum test, *P* = 7.6e‒7), while those with *GNAS* copy number gain showed increased GNAS at both mRNA and protein levels (Wilcoxon rank-sum test, *P* = 0.014 and *P* = 0.021) (Fig. 3d; Supplementary information, Fig. S3a, b).

*GNAS* mutations in PitNET patients have been linked to a gain of function in G protein-coupled receptor (GPCR) signaling pathways,^26^ although the specific downstream impacts remain unknown. Compared with the WT group, we found that genes involved in growth hormone (GH) synthesis, secretion, and action pathways (e.g., *GH1* and *GH2*) were upregulated at the mRNA, protein, and phosphorylation levels (q < 0.05) in the *GNAS* mutant group (Fig. 3e, f; Supplementary information, Fig. S3c, d and Table S3). More specifically, we identified the upregulation and phosphorylation (ADCY5_S666 and ADCY6_S576) of adenylate cyclase (AC), along with the expression of PKAs in the *GNAS* mutant PIT1 lineage tumors. We further focused on the components in the GNAS-PKA downstream pathways. Combining with the known mechanism, we speculated that PKAs might promote the phosphorylation of CREBBP and subsequent accumulation of the CREB complex (CREB1 and ATF2), ultimately leading to hypersecretion of GH through PIT1 activation. Likewise, we also infer that the hypersecretion of GH in these samples might affect the levels of SHC (i.e., SHC1 and SHC3) and STAT5B, as well as AKT1 and PKC protein activities (i.e., PRKCA and PRKCB), which are known to promote cell growth and metabolism.

Amplification of 20q has been reported in PitNET,^27^ while the *cis* and *trans* effects of 20q amplification and *GNAS* CNA (located at 20q) remain unclear. Gene set enrichment analysis (GSEA) of transcriptomics/proteomics datasets by Spearman’s correlation showed upregulation in proliferation-related pathways, such as cell cycle and DNA replication pathways, in patients with *GNAS* copy number gain (FDR < 0.05) (Fig. 3h; Supplementary information, Fig. S3e, f and Table S3). Furthermore, the *GNAS* copy number gain group had higher multigene proliferation score (MGPS) and clinical tumor volume as compared with the WT group (Wilcoxon test, *P* < 0.05) (Fig. 3i). However, the correlation was non-significant in tests with GH PitNETs alone.^28^ To determine the proliferation characteristics of the PIT1 lineage driven by *GNAS* copy number gain, we systematically characterized the signal cascade related to cell cycle and DNA synthesis. Among cell cycle-related molecules, PRKDC and CDK6 were the top two proteins positively correlated with *GNAS* copy number (Fig. 3j; Supplementary information, Fig. S3g). Chemical inhibition or knockdown of GNAS has been shown to decrease the expression of cyclin proteins such as cyclin D, which is closely related to CDK6.^29,30^ This combined evidence suggested that CDK6 could contribute to the enhanced proliferation rate of PIT1 PitNETs as a result of *GNAS* copy number gain. It was also noteworthy that Rb mRNA level was positively correlated with GNAS copy number, mRNA and protein levels (Spearman’s correlation: *GNAS* copy number, R = 0.26, *P < 0.05; *GNAS* mRNA, R = 0.30, **P < 0.01; GNAS protein, R = 0.26, **P < 0.01) (Supplementary information, Fig. S3h). In addition, Rb phosphorylation levels at the RB1_S37 site were significantly correlated with GNAS at the mRNA and protein levels (Spearman’s correlation: GNAS mRNA, R = 0.23, *P < 0.05; GNAS protein, R = 0.33, ***P < 0.001). Finally, our data showed that E2F and CDK2 were downregulated, which might lead to the upregulation of ORC family members. The upregulation of the ORC family, RFC family, and PCNA in patients with *GNAS* copy number gain likely led to the elevated DNA biosynthesis and the enhanced tumor cell proliferation (Fig. 3j).

To further confirm the impacts of *GNAS* copy number gain, we performed immunohistochemistry (IHC) for GNAS and CDK6 and calculated IHC staining scores (H-scores). H-scores of GNAS and CDK6 were divided into high and low H-score groups based on the median score, respectively. As expected, the proportion of CDK6 high H-score cells was greater in the GNAS high H-score group than in the GNAS low H-score group in both the PIT1 lineage PitNETs and all PitNETs (Fisher’s exact test: PIT1 lineage PitNETs, *P* = 0.026; all PitNETs, *P* = 0.031) (Fig. 3k, l; Supplementary information, Fig. S3i).

In conclusion, these findings illustrate the diverse impacts of genomic events in the *GNAS* gene, such as mutations that drive hormone hypersecretion. Moreover, the finding that *GNAS* copy number gain can markedly enhance tumor cell proliferation implied that an inhibitor therapy targeting CDK6 may be effective for PIT1 lineage patients harboring *GNAS* copy number gain.

### Multi-omics classification of PitNETs

To comprehensively explore the phenotypic and genotypic PitNET diversity in this cohort, classification by consensus clustering^31^ was performed with the combined transcriptomics, proteomics, and phosphoproteomics data. This analysis identified seven proteomic (Supplementary information, Fig. S4a), five transcriptomic (Supplementary information, Fig. S4b), and seven phosphoproteomic (Supplementary information, Fig. S4c) clusters among the PitNETs (Supplementary information, Table S4), which were subsequently named according to their similarities to clinicopathological subtypes and predominant pathway associations.

**Fig. 4:**
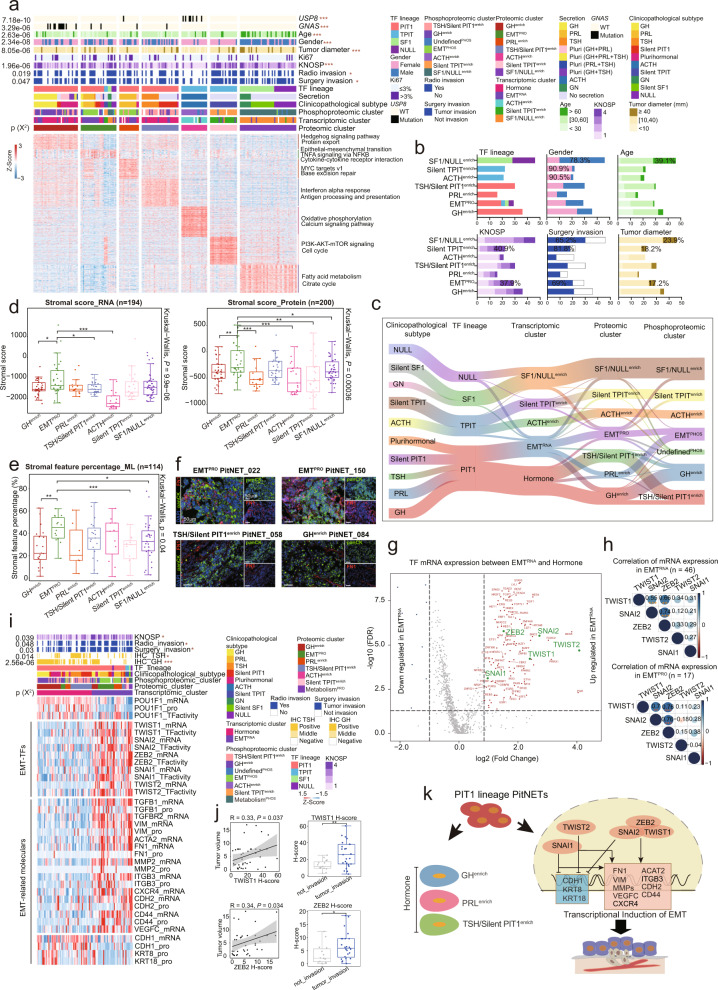
Molecular subtypes of PitNETs based on proteogenomic analysis and association studies. **a** Heatmap illustrating the characterization of seven proteomic clusters. Each column represents a patient sample and rows indicate proteins. The color of each cell shows the *z* score of the protein in that sample. PitNET classification, hormone secretion status, invasion status, clinical features, and mutation status annotations are shown above the heatmap. The chi-square test was used to evaluate the association of proteomic clusters with the 9 variables on the heatmap (*P < 0.05, **P < 0.01, ***P < 0.001). Single-sample Gene Set Enrichment Analysis (ssGSEA) based on proteomics data was also applied to identify the dominant pathway signatures in each proteomic cluster. **b** Summary of the variables with significant differences among the seven proteomic clusters. The percentage represents the proportion of the population. **c** Sankey diagram depicting the result of integrative multi-omics analysis, showing the flow of cluster assignments across multiple classification of PitNETs. **d** Boxplots depicting the distribution of stromal scores inferred by ESTIMATE based on the RNA data (left) and protein data (right) among tumors of the seven proteomic clusters. Kruskal-Wallis test was used to test if any of the differences among the subgroups were statistically significant. The Wilcoxon rank-sum test was used to estimate the difference between two subgroups, *P < 0.05, **P < 0.01, ***P < 0.001. **e** Boxplot depicting the distribution of stromal scores based on stromal feature percentage_ML among tumors of the seven proteomic clusters. Kruskal-Wallis test was used to test whether any of the differences among the subgroups were statistically significant. Wilcoxon rank-sum test was used to estimate the difference between two subgroups, *P < 0.05, **P < 0.01, ***P < 0.001. **f** Representative IF staining of pan-cytokeratin (panCK) and fibronectin1 (FN1) in EMT^PRO^ and non-EMT^PRO^ clusters. Scale bar, 50 μm. **g** Volcano plot showing differential mRNA expression of TFs between EMT^RNA^ and Hormone clusters (the horizontal axis is log_2_(fold change), and the vertical axis is –log_10_ FDR). The upregulated TFs in EMT^RNA^ are highlighted in red and EMT-TFs are highlighted in green. **h** Correlation heatmaps showing the correlation among the mRNA expression of five EMT-TFs in EMT^RNA^ and EMT^PRO^ clusters. Spearman’s correlation, **P < 0.01, ***P < 0.001. **i** Taking POU1F1 as the positive control, heatmap showing the molecules significantly differentially expressed between EMT^RNA^ and Hormone clusters at the mRNA, protein, and TF activity levels, including EMT-TFs and EMT-related markers. **j** IHC staining validated the correlation between EMT-TFs and tumor invasion. Scatterplots showing the correlation of H-scores of TWIST1 and ZEB2 with tumor volume (Spearman’s correlation). The boxplots show the association of H-scores of TWIST1 and ZEB2 with surgery invasion status (Wilcoxon rank-sum test). **k** Summary of the multi-omics classification of the PIT1 lineage.

At the protein level, the seven proteomic clusters included GH^enrich^, EMT^PRO^, PRL^enrich^, TSH/silent PIT1^enrich^, ACTH^enrich^, silent TPIT^enrich^, and SF1/NULL^enrich^ (Fig. 4a). Pathway enrichment analysis (see Materials and methods; Supplementary information, Table S4) showed that the Hedgehog signaling pathway was differentially upregulated in GH^enrich^, and MYC targets v1 was upregulated in PRL^enrich^ (Fig. 4a). TSH and silent PIT1 were co-clustered and enriched for pathways such as interferon-ɑ response, and antigen processing and presentation. In addition, the SF1 lineage and NULL PitNETs clustered together, forming the SF1/NULL^enrich^ cluster, which showed upregulation in metabolism-related pathways, including fatty acid metabolism and the citrate cycle. Moreover, males were more prevalent (78.3%), average age was higher (> 60, 39.1%), and tumor diameter was larger (≥ 40 mm, 23.9%) in the SF1/NULL^enrich^ cluster compared to other clusters (Fig. 4b). Notably, the TPIT lineage was divided into two smaller clusters, ACTH^enrich^ and silent TPIT^enrich^, at the protein level, which was consistent with clinicopathological subtypes (Supplementary information, Fig. S4d, e). The ACTH^enrich^ cluster was enriched for *USP8* mutations and both ACTH^enrich^ and silent TPIT^enrich^ had an extremely high proportion of females (90.5% and 90.9%, respectively) (Fig. 4b). In addition, ACTH^enrich^, silent TPIT^enrich^, and SF1/NULL^enrich^ clusters were all associated with higher MGPS (Kruskal-Wallis test, *P* = 7.4e‒06) (see Materials and methods; Supplementary information, Fig. S4f), which was aligned well with the upregulation of proliferation and energy metabolism pathways in these three clusters.

To further characterize the proteogenomic classification of PitNETs, we performed integrative analysis of the ten clinicopathological subtypes, three TF lineages and NULL, five transcriptomic clusters, seven proteomic clusters, and seven phosphoproteomic clusters for PitNETs. Interestingly, the ACTH^enrich^, silent TPIT^enrich^, and SF1/NULL^enrich^ clusters identified using proteomics data were highly consistent with clusters identified using transcriptomics and phosphoproteomics data (Fig. 4c). Furthermore, a cluster of PitNETs was also identified with clear EMT characteristics at the transcriptomics, proteomics and phosphoproteomics levels.

### An invasive cluster characterized by EMT was identified within the PIT1 lineage

At the protein level, we found that hemostasis-related and EMT-related molecules,^32–34^ including GP1BB, FGB, MMP8, FN1, and ITGB3, were highly expressed in EMT^PRO^, compared with other proteomic clusters (Supplementary information, Table S4). The pathways related to EMT, TNFA signaling via NFκB, and cytokine‒cytokine receptor interaction were all upregulated in the EMT^PRO^ cluster (Fig. 4a), which covered eight of the ten clinicopathological subtypes, excluding ACTH and silent SF1 (Supplementary information, Fig. S4d). Strikingly, EMT^PRO^ showed strong invasiveness (Fig. 4b), with a high level of KNOSP (grade = 4, 37.9%), surgery invasion (69%), and tumor diameter (≥ 40 mm, 17.2%).

Given the non-negligible role of EMT in cancer metastasis,^35,36^ we next used the ESTIMATE algorithm^37^ to deconvolute the contribution of stromal cells in the tumors based on transcriptomics data (Stromal score_RNA) and proteomics data (Stromal score_Protein). EMT^PRO^ showed overall higher stromal scores in both Stromal score_RNA (Kruskal-Wallis test, *P* = 9.9e‒06) and Stromal score_Protein (Kruskal-Wallis test, *P* = 3.6e‒04) (Fig. 4d). Hematoxylin and eosin (HE) staining was also processed to evaluate the proportion of tumor cells that featured stromal morphology with quantification by QuPath bioimage analysis (Stromal feature percentage_ML, see Materials and methods) and confirmed that the EMT^PRO^ cluster had the highest proportion of cells with a stromal phenotype (Kruskal-Wallis test, *P* = 0.04) among all the proteomic clusters (Fig. 4e; Supplementary information, Fig. S4g). To further investigate the EMT status of tumor cells in each of the seven proteomic clusters, immunofluorescence (IF) co-staining was performed to detect the epithelial marker, pan-cytokeratin (panCK), and mesenchymal marker, fibronectin1 (FN1), in a large subset of tumors. The IF results showed significantly higher percentage of areas with co-staining of panCK and FN1 in EMT^PRO^ cluster than in other clusters (Fig. 4f; Supplementary information, Fig. S4h). All these pieces of evidence supported that EMT^PRO^ cluster was characterized by tumor cells with EMT status.

EMT-inducing transcription factors (EMT-TFs)^38^ are those confirmed as key drivers of the EMT-phenotype. We compared TF mRNA expression level between the two transcriptomic clusters in the PIT1 lineage (Fig. 4g), which confirmed that five EMT-TFs were significantly upregulated in EMT^RNA^ including: *SNAI1* (FC = 1.97, FDR = 0.000074), *SNAI2* (FC = 6.63, FDR = 2.3e‒06), *ZEB2* (FC = 3.04, FDR = 2.1e‒06), *TWIST1* (FC = 5.3, FDR = 5.93e‒05), and *TWIST2* (FC = 17, FDR = 2.02e‒05). The remaining PIT1 lineage cluster was designated Hormone due to upregulation of hormone secretion proteins. Furthermore, the mRNA expression patterns of the five EMT-TFs were significantly positively correlated in both the EMT^RNA^ (Spearman’s correlation, R = 0.55‒0.74, *P* < 0.01) and EMT^PRO^ (Spearman’s correlation, R = 0.70‒0.76, *P* < 0.01) clusters (Fig. 4h). Notably, the levels of transcriptional activity of the five EMT-TFs were higher in the EMT^RNA^ cluster compared with those in the Hormone cluster (Fig. 4i). Similarly, EMT-related molecules^35,39^ including CDH2, VIM, CD44, FN1, and ITGB1 (mesenchymal markers) were upregulated in the EMT^RNA^ cluster, while CDH1, KRT8, and KRT18 (epithelial markers) were downregulated in the transcriptomics and proteomics datasets (Fig. 4i) further confirming the EMT status of this cluster.

In addition, IHC staining of TWIST1 and ZEB2 in our cohort verified that EMT-TFs were activated in EMT^RNA^ (Supplementary information, Fig. S4i). There were significant positive correlations between the H-scores of TWIST1 and ZEB2 and their corresponding tumor volumes (Spearman’s correlation: TWIST1, R = 0.33, *P* = 0.037; ZEB2, R = 0.34, *P* = 0.034), and the high H-scores of TWIST1 and ZEB2 were associated with surgery invasion (Wilcoxon rank-sum test, TWIST1: *P* = 0.0083; ZEB2: *P* = 0.025) in the PIT1 lineage (Fig. 4j).

In summary, integrated proteogenomic characterization of PitNETs identified a previously unrecognized, highly invasive cluster defined by EMT in PitNETs, primarily containing PIT1 lineage tumors (Fig. 4k).

### Proteogenomics data revealed three modes of EGFR activation in the TPIT lineage

EGFR is associated with a variety of human cancers, including head and neck squamous cell carcinoma and lung adenocarcinoma,^40–42^ and has been proposed as a therapeutic target in ACTH PitNETs.^11,43^ Here, we found that the levels of EGFR mRNA expression, protein abundance, and phosphorylation modifications were higher in the TPIT lineage than in other tumors (Supplementary information, Fig. S5a‒c), which expanded the previous perception that EGFR was highly expressed in ACTH PitNETs.^11^ Subsequent analysis of EGFR-related pathways defined three groups that showed diverse mechanisms of EGFR activation, including ACTH tumors with *USP8* mutation (ACTH_*USP8* mutant), ACTH tumors without *USP8* mutation (ACTH_ *USP8* WT), and silent TPIT tumors (Supplementary information, Fig. S5b, Table S5). In the ACTH_*USP8* mutant group, our data supported the known mechanism that *USP8* gain-of-function mutations rescue EGFR from ubiquitination, leading to the enhanced EGFR activity, and further promoting POMC biosynthesis (Supplementary information, Fig. S5c, d).^15,16^ In the ACTH_ *USP8* WT group, the average mRNA expression of EGFR ligands (i.e., AREG, TGFA, EGF, BTC, EPGN, HBEGF, and NRG4) was significantly higher than in other groups (Kruskal-Wallis test, *P* = 0.009) (Supplementary information, Fig. S5e) and positively correlated with peptide hormone biosynthesis (Spearman’s correlation, R = 0.4, *P* = 0.0079) and serum ACTH level (Spearman’s correlation, R = 0.34, *P* = 0.026) (Supplementary information, Fig. S5f, g). In silent TPIT tumors, EGFR T693 phosphorylation showed a significant enrichment (Kruskal-Wallis test, *P* = 0.0087), and EGFR downstream pathways or components, including PI3K-AKT-mTOR (Spearman’s correlation, R = 0.38, *P* = 0.0093), MAPK (Spearman’s correlation, R = 0.33, *P* = 0.023) and the cell cycle pathways were also enriched (Spearman’s correlation, R = 0.19, *P* = 0.2) (Supplementary information, Fig. S5h‒j), suggesting that EGFR T693 phosphorylation may lead to activation of these pathways. Furthermore, IHC staining of EGFR revealed that its expression was higher in the TPIT lineage than in non-TPIT lineages, and a higher positive staining rate of EGFR T693 phosphorylation was found in silent TPIT tumors as compared with ACTH tumors (Supplementary information, Fig. S5k). Based on these results, we summarized the potential therapeutic options for each of the three modes (Supplementary information, Fig. S5l).

In addition to the finding of the effects of EGFR on POMC biosynthesis, we also explored whether and which TFs were involved in the biological features of ACTH vs silent TPIT subtype. Based on mRNA levels and the predicted transcriptional activity, we identified four TFs, ASCL1, AHRR, CUX2, and KLF15, that were potentially involved in POMC biosynthesis or ACTH secretion using multi-omics data (Supplementary information, Fig. S6a, b). Among them, ASCL1 overexpression and activation was reported to lead to excessive ACTH secretion.^44^ However, further study is necessary to fully understand the different mechanisms of EGFR/ASCL1-POMC in ACTH and EGFR-PI3K-AKT-mTOR in silent TPIT.

To sum up, these analyses suggested three potential modes of EGFR activation in TPIT lineage PitNETs which could result in different molecular characteristics. In addition, four TFs were identified that may be involved in ACTH secretion and regulation, and could possibly serve as novel therapeutic targets.

### VEGF and hypoxia signaling were activated in the SF1 lineage and NULL tumors

In light of our above multi-omics-based subtyping results that the SF1 lineage and NULL tumors showed similar expression patterns, especially for glycolysis and mitochondrial citrate cycle enzymes (e.g., IDH1, IDH2, IDH3A, IDH3B and IDH3G) (Supplementary information, Fig. S7a), which leads to their reassignment as the SF1/NULL^enrich^ cluster, we next conducted enrichment analysis based on PROGENy scores to assess whether 14 well-established cancer-relevant pathways (as defined by Schubert et al. and Holland et al.)^45,46^ were activated in our cohort of 200 PitNETs (Supplementary information, Fig. S7b and Table S6). The results showed that the hypoxia (Kruskal-Wallis test, *P* = 0.00021) and VEGF (Kruskal-Wallis test, *P* = 2.1e‒11) pathways were enriched in both the SF1 lineage and NULL tumors compared with other PitNETs (Supplementary information, Fig. S7c). Furthermore, we observed that signature genes of hypoxia (HIF1A and HIF1B) and angiogenesis (VEGFA, VEGFR2 and PECAM1) were overexpressed in the SF1 lineage and NULL tumors (Supplementary information, Fig. S7d). Interestingly, the mRNA expression levels of VEGFA and VEGFR2 were significantly positively correlated with inferred VEGF pathway activity (Spearman’s correlation: VEGFA, R = 0.30, *P* = 0.027; VEGFR2, R = 0.35, *P* = 0.011) (Supplementary information, Fig. S7e). To advance our understanding of the biological mechanisms of these tumors, VEGF signaling and angiogenesis-related genes were analyzed in the multi-omics datasets (Supplementary information, Table S6). We found that downstream pathways of VEGF signaling, e.g. RAS/RAF/MEK/ERK and PI3K-AKT, were activated in both the SF1 lineage and NULL tumors at the mRNA, protein, and phosphoprotein levels (Supplementary information, Fig. S7d, f).

Consistent with the results obtained from multi-omics data, IHC staining of VEGFR2 showed its higher expression in the SF1 lineage (GN and silent SF1) and NULL tumors than in other PitNETs (Kruskal-Wallis test, VEGFR2 H-score among TF lineages, *P* = 0.00012; VEGFR2 H-score among clinicopathological subtypes, *P* = 0.0021) (Supplementary information, Fig. S7g, h). These collective results implied that the SF1 lineage and NULL tumors have similar molecular features and can be clustered together.

Taken together, the above data show that hypoxia and VEGF signaling pathways are uniquely upregulated in both the SF1 lineage and NULL tumors, suggesting that angiogenesis inhibitors targeting VEGFR2 may serve as effective therapeutic approaches for these patients.

### Characterization of immune infiltration in PitNETs

**Fig. 5:**
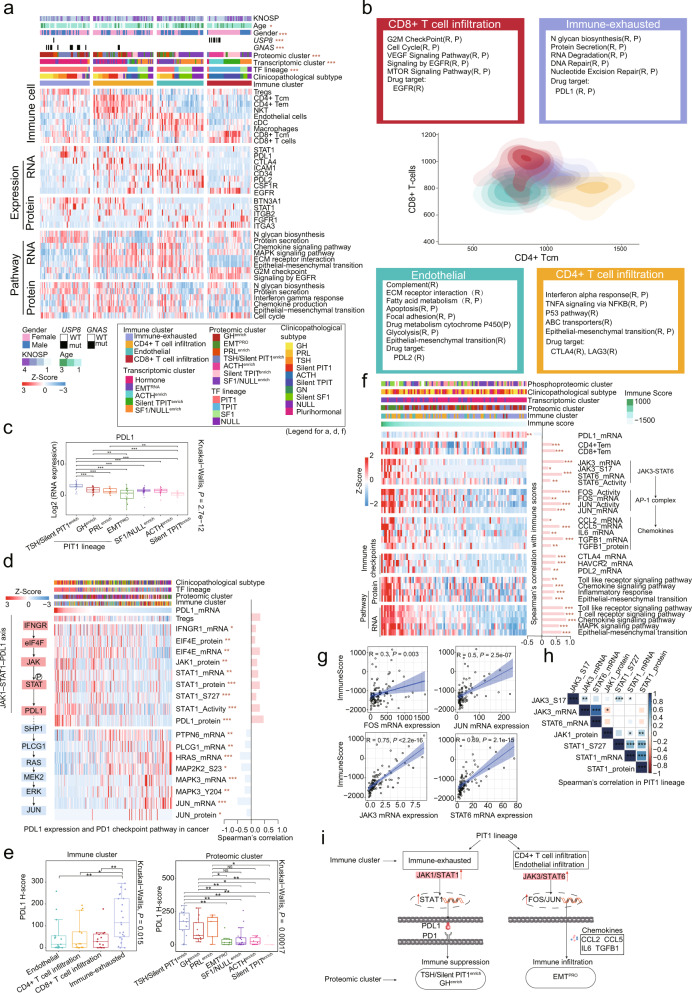
Immune landscape in PitNETs. **a** The four immune clusters identified by consensus clustering showing cell-type features, immune checkpoints, and ssGSEA pathways. Differential expression between tumors of one immune cluster vs the rest at the mRNA and protein levels (Wilcoxon rank-sum test, *P* < 0.05) and the corresponding enriched pathways (Wilcoxon rank-sum test, *P* < 0.05) were shown. Chi-square test was used to test the association of immune clusters with the 9 variables on the heatmap (*P < 0.05, ***P < 0.001). **b** Contour plot of two-dimensional density based on CD8^+^ T cells scores (*y*-axis) and CD4^+^ Tcm scores (*x*-axis) for different immune clusters. For each immune cluster, key upregulated pathways, and significant drug targets (Wilcoxon rank-sum test, *P* < 0.05) identified based on RNA-seq (R) and proteomics (P) are reported in the annotation boxes. **c** Boxplot of *PDL1* mRNA among the seven proteomic clusters. Kruskal-Wallis test was used to test whether any of the differences among the subgroups were statistically significant. Wilcoxon rank-sum test was used to estimate the significance of two subgroups, **P < 0.01; ***P < 0.001. **d** PD1-PDL1 signaling pathway-related genes were highly correlated with *PDL1* mRNA expression at the mRNA, protein, and phosphoprotein levels in all PitNETs. The bar chart on the right shows Spearman’s correlation coefficient with *PDL1* mRNA expression (*P < 0.05, **P < 0.01, ***P < 0.001). **e** Boxplots showing the PDL1 H-score among proteomic clusters and immune clusters. Kruskal-Wallis test was used to test whether any of the differences among the subgroups were statistically significant. Wilcoxon rank-sum test was used to estimate the significance of two subgroups, *P < 0.05; **P < 0.01, NS, not significant. **f** Spearman’s correlations (*P* < 0.05) between the ESTIMATE immune score and proteogenomic profiles of immune infiltration, chemokines, immune checkpoints, and pathways in the PIT1 lineage. **g** Scatterplots showing the Spearman’s correlation of the immune score with the mRNA expression of FOS, JUN, JAK3, and STAT6. **h** Spearman’s correlation among JAK1, JAK3, STAT6, and STAT1 at the mRNA, protein, and phosphorylation levels in the PIT1 lineage (*P < 0.05, **P < 0.01, ***P < 0.001). **i** Diagram depicting the mechanism of the distinct immune clusters within the PIT1 lineage.

We next performed cell type deconvolution using xCell^47^ analysis of transcriptomics data to infer the proportion of different cell types in the tumor microenvironment (Fig. 5a; Supplementary information, Table S7). Consensus clustering based on inferred cell proportions identified four sets of tumors with distinct immune and stromal features: Immune-exhausted, CD4^+^ T cell infiltration, Endothelial, and CD8^+^ T cell infiltration (Fig. 5a).

The CD8^+^ T cell infiltration cluster, containing the ACTH^enrich^ and silent TPIT^enrich^ clusters, was characterized by the presence of multiple immune cell types, including central memory CD8^+^ T cells and CD8^+^ T cells (Fig. 5a; Supplementary information, Fig. S8a). Moreover, the CD8^+^ T cell infiltration cluster showed upregulation of EGFR signaling and cell cycle pathways (Fig. 5a, b). The Endothelial cluster was characterized by antigen presenting cells such as macrophages and cDCs, with upregulation of CSF1R, CD34, and PDL2 at the mRNA level and FGFR1 at the protein level (Fig. 5a, b). In the CD4^+^ T cell infiltration cluster, the immunosuppressive mediator CTLA4 was upregulated (CD4^+^ T cell infiltration cluster vs other immune clusters: Wilcoxon rank-sum test, *P* = 0.008), suggesting that these tumors might be responsive to immune checkpoint-related therapeutic options (Fig. 5a, b). The Immune-exhausted cluster, consisting of the TSH/silent PIT1^enrich^ and GH^enrich^ proteomic clusters, was mainly distributed in the PIT1 lineage (Supplementary information, Fig. S8a, b), and characterized by higher scores of Treg cells and upregulation of PDL1 (CD274) (Kruskal-Wallis test, *P* = 1e‒05) based on transcriptomics data (Fig. 5a; Supplementary information, Fig. S8c).

Indeed, PDL1 mRNA expression was significantly upregulated in the TSH/silent PIT1^enrich^ and GH^enrich^ proteomic clusters (Kruskal-Wallis test, *P* = 2.7e‒12) (Fig. 5c), as well as in the TSH, silent PIT1, and GH clinicopathological subtypes (Kruskal-Wallis test, *P* = 7.1e‒08) (Supplementary information, Fig. S8d). Given its role in immune suppression,^48^ we further explored the correlation between PDL1 mRNA expression and JAK1-STAT1-PDL1-related molecules, including IFNGR1, EIF4E, JAK1, and STAT1 in PitNETs (Fig. 5d). As expected, these molecules were significantly positively correlated at the mRNA, protein, and phosphoprotein levels. In addition, we observed that TCR signaling-related genes, including *PTPN6*, *PLCG1*, and *JUN*, were significantly negatively correlated with *PDL1* mRNA expression (Fig. 5d), which is consistent with the PD1-PDL1 immune checkpoint mechanism.^49^ The multi-omics data suggested that activation of the JAK1-STAT1-PDL1 axis could inhibit antitumor immune response through adaptive immune resistance based on the high transcription levels of PDL1 observed in the TSH/silent PIT1^enrich^ and GH^enrich^ clusters, suggesting that anti-PDL1 therapies might warrant exploration in these tumors. Further validation of PDL1 by IHC staining in our PitNET cohort revealed that it was expressed at higher levels in some clinicopathological subtypes, including TSH, silent PIT1, and GH, than in other subtypes (Supplementary information, Fig. S8e). The PDL1 H-score was significantly higher in the TSH/silent PIT1^enrich^ and GH^enrich^ clusters (proteomic subtyping) and Immune-exhausted cluster (immune subtyping), which is consistent with findings obtained from multi-omics data (Fig. 5e).

In the PIT1 lineage, we found that PDL1 was upregulated in the TSH/silent PIT1^enrich^ and GH^enrich^ clusters, consistent with their lower immune scores. In contrast, other clusters in the PIT1 lineage, such as the EMT^PRO^ cluster, had higher immune scores (Supplementary information, Fig. S8f) and low PDL1 expression. Interestingly, the expression of another JAK-STAT axis, JAK3-STAT6-FOS/JUN, was highly correlated with immune score (Fig. 5f; Supplementary information, Fig. S8g). STAT6 has been reported to regulate FOS and JUN, further contributing to tumor progression.^50–52^ In addition, the FOS and JUN TFs regulate downstream chemokines, such as CCL2, CCL5, IL6, and TGFB1,^53^ all of which showed significantly positive correlation with immune scores at the mRNA and protein levels (Fig. 5f, g; Supplementary information, Fig. S8g). These findings led us to propose that increased immune infiltration caused by chemokine expression in the EMT^PRO^ cluster was likely regulated by the JAK3-STAT6-FOS/JUN axis. In the multi-omics data, JAK1/STAT1 showed a moderate negative correlation with JAK3/STAT6 in both the PIT1 lineage tumors and all PitNETs (Fig. 5h; Supplementary information, Fig. S8h).

Taken together, these data showed unexpected bidirectional regulation in the PIT1 lineage, including immune suppression mechanisms in the TSH/silent PIT1^enrich^ and GH^enrich^ clusters that might be responsive to checkpoint (PDL1 and PD1) inhibitors, and immune infiltration mechanisms in the EMT^PRO^ cluster that could be potentially targeted with immunotherapies (Fig. 5i).

### Expression of available drug targets among proteomic clusters

To expand the potential treatment options for PitNET patients using these multi-omics data, we next evaluated the expression levels of targets of FDA-approved drugs including dopamine receptor 2 (DRD2) and somatostatin receptors (SSTR2/SSTR5), as well as O-6-methylguanine-DNA methyltransferase (MGMT) involved in DNA repair, which is known to affect temozolomide efficiency.^6,54^ Somatostatin agonists are the predominant treatment for GH PitNETs and are reported to be effective against ACTH PitNETs.^3^ Transcriptomics data indicated that *SSTR2* (Kruskal-Wallis test, *P* < 2.2e‒16) and *SSTR5* (Kruskal-Wallis test, *P* < 2.2e‒16) were elevated in GH PitNETs as well as in the TSH/silent PIT1^enrich^ proteomic cluster (Supplementary information, Fig. S9a‒c). We further found that *MGMT* mRNA was downregulated in the PRL^enrich^, TSH/silent PIT1^enrich^, and silent TPIT^enrich^ proteomic clusters compared with other PitNETs (Wilcoxon rank-sum test: PRL^enrich^, *P* = 0.001; TSH/silent PIT1^enrich^, *P* = 0.0002; silent TPIT^enrich^, *P* = 0.0005) (Supplementary information, Fig. S9a, d). *DRD2* overexpression has been reported in PRL and GN PitNETs,^55^ which aligned with our results showing significant upregulation of *DRD2* mRNA in PRL^enrich^ (Wilcoxon rank-sum test, *P* = 2.37e‒05) and SF1/NULL^enrich^ (GN and NULL) clusters (Wilcoxon rank-sum test, *P* = 1.21e‒05) (Supplementary information, Fig. S9a, e).

### Validation of markers in an independent cohort of 750 PitNETs

**Fig. 6:**
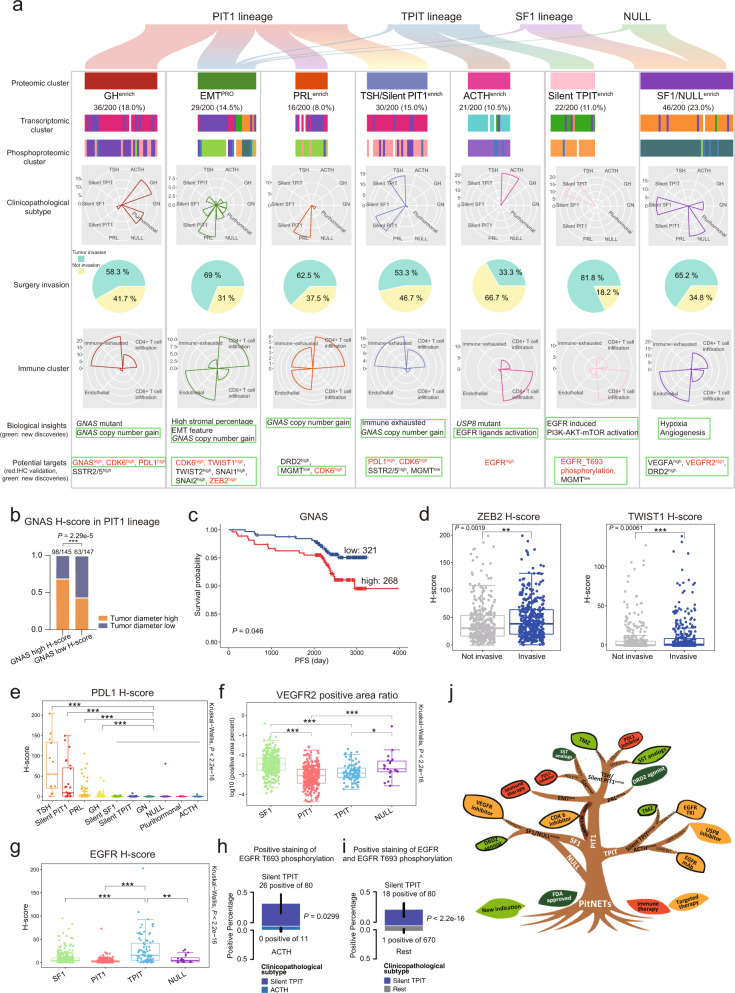
Summary of molecular characteristics based on proteomic clusters in 200 PitNETs and validation of potential targets in an independent cohort. **a** Graphical summary showing the major molecular findings of 200 PitNETs: heatmap showing unbiased consensus clustering of proteomic clusters, transcriptomic clusters and phosphoproteomic clusters; radar maps showing different proportions of seven proteomic clusters in clinicopathological subtypes and immune clusters; pie charts represent surgery invasion; biological insights and potential targets are listed at the bottom. Novel prognostic markers and therapeutic targets were marked by green boxes in the last two lines. IHC validation molecules are marked with red font. **b** Bar plot showing the proportion of high tumor diameter between GNAS high H-score group and GNAS low H-score group in the cohort of 750 PitNETs. The significance was calculated by Fisher’s exact test. **c** GNAS staining is correlated with PFS in the cohort of 750 PitNETs (log-rank test). **d** Boxplots describing the high H-scores of ZEB2 and TWIST1 in tumor invasive group compared with non-invasive group in 750 PitNET cohort (Wilcoxon rank-sum test). **e** IHC staining of PDL1 in an independent cohort of 750 PitNETs. Boxplot showing the H-score of PDL1 in TSH, silent PIT1, PRL, GH and other clinicopathological subtypes, respectively (Wilcoxon rank-sum test, ***P < 0.001). Kruskal-Wallis test was used to test whether any of the differences among the subgroups were statistically significant. **f** IHC staining of VEGFR2 in an independent cohort of 750 PitNETs. Boxplot showing the percentage of tumor tissues with positive staining among TF lineages. Kruskal-Wallis test was used to test whether any of the differences among the subgroups were statistically significant. Wilcoxon rank-sum test was used to estimate the significance of two subgroups, *P < 0.05, ***P < 0.001. **g** IHC staining of EGFR in an independent cohort of 750 PitNETs. Boxplot showing the H-score among TF lineages. Kruskal-Wallis test was used to test whether any of the differences among the subgroups were statistically significant. Wilcoxon rank-sum test was used to estimate the significance of two subgroups, **P < 0.01, ***P < 0.001. **h** Bar plot showing the proportion of EGFR T693 phosphorylation-positive staining in silent TPIT and ACTH subtypes based on Fisher’s exact test. **i** Bar plot showing the proportion of both EGFR and EGFR T693 phosphorylation-positive staining in silent TPIT compared to other subtypes based on Fisher’s exact test. **j** The PitNET tree shows that the main TF lineages (PIT1, TPIT, and SF1) and NULL tumors can be further divided into seven proteomic clusters (GH^enrich^, EMT^PRO^, PRL^enrich^, TSH/silent PIT1^enrich^, ACTH^enrich^, silent TPIT^enrich^, and SF1/NULL^enrich^). Dark green leaves represent drugs with FDA approval for use in the specific reported clinicopathological subtypes, while light green leaves represent new indications of FDA-approved drugs with potential efficacy in patients based on proteomic clusters. Red leaves represent immune therapies and orange leaves represent potential targeted therapies. Leaves with black outlines are newly discovered in our study. Abbreviations in PitNET tree: TMZ, temozolomide; TKI, tyrosine kinase inhibitor; mAB, monoclonal antibody; MGMT, O-6-methylguanine-DNA methyltransferase; SST, somatostatin.

Our study identified seven robust tumor clusters and the representative molecular characteristics of each proteomic cluster were summarized in Fig. 6a and Supplementary information, Table S8. To evaluate whether the results observed in this study were robust beyond the initial cohort, we verified the findings in an independent cohort of 750 PitNETs, with follow-up data for 78% of patients (Supplementary information, Table S9). The overall average follow-up duration was 85 months. The proportion of GNAS high H-score was significantly greater in the high tumor diameter group than in the low tumor diameter group (Fisher’s exact test, *P* = 2.29e‒5), with a high GNAS H-score suggesting poor prognosis (log-rank test, *P* = 0.046) (Fig. 6b, c). These results were consistent with findings obtained in the initial cohort of 200 PitNETs. Examination of the EMT-TFs identified in the 200 PitNET cohort (Fig. 4j) indicated that ZEB2 and TWIST1 had higher H-scores in the invasive group in the validation cohort of 750 PitNETs (Wilcoxon rank-sum test, *P* = 0.0019 and *P* = 0.00061) (Fig. 6d). Moreover, in the 750 PitNETs, PDL1 showed higher H-scores in the TSH and silent PIT1 clinicopathological subtypes (Kruskal-Wallis test, *P* < 2.2e − 16) (Fig. 6e), supporting our finding of PDL1 overexpression in the Immune-exhausted cluster in the 200 PitNET cohort (Fig. 5c). IHC staining for VEGFR2 in the 200 and 750 PitNET cohorts consistently showed its higher expression in the SF1 and NULL tumors (Wilcoxon rank-sum test, *P* = 0.00012 in the 200 PitNET cohort and *P* < 2.2e‒16 in the 750 PitNET cohort) (Fig. 6f; Supplementary information, Fig. S7g). In the 750 PitNET validation cohort, EGFR was uniquely overexpressed in the TPIT lineage compared with other lineages, which is also consistent with results of the 200 PitNET cohort (Fig. 6g; Supplementary information, Fig. S5k). IHC staining for EGFR T693 phosphorylation confirmed that the proportion of IHC-positive cases was significantly higher in the silent TPIT (26 of 80) subtype than in ACTH (0 of 11) within the TPIT lineage (Fisher’s exact test, *P* = 0.0299) (Fig. 6h). Additionally, the percentage of patients with positive staining for both EGFR and EGFR T693 phosphorylation was significantly higher in the silent TPIT (18 of 80) subtype than in other lineages (1 of 670) (Fisher’s exact test, *P* < 2.2e‒16) (Fig. 6i).

In conclusion, the prognostic markers and therapeutic targets GNAS, ZEB2, TWIST1, PDL1, VEGFR2, EGFR, and EGFR T693 phosphorylation identified in our initial 200 PitNET cohort were validated in the corresponding subtypes of the 750 PitNET cohort. These collective findings are summarized in a PitNET tree, which shows an updated molecular classification in which patients are clearly stratified into seven clusters for potential therapeutic options (Fig. 6j).

## Discussion

In this study, genomics, transcriptomics, proteomics, and phosphoproteomics datasets were generated as a public resource from a retrospective cohort of 200 PitNETs and 7 APGs collected at a single center. We identified three genomic events, including *GNAS* mutation, *GNAS* copy number gain and *USP8* mutation, as well as several other findings related to updated classification and stratified therapies, through integrative analysis of transcriptomics, proteomics and phosphoproteomics datasets. To the best of our knowledge, this study represents the largest integrated proteogenomic study of PitNET to date, spanning all ten clinicopathological subtypes. In addition, several therapeutic targets (e.g., GNAS, CDK6, TWIST1, ZEB2, PDL1, EGFR, EGFR T693 phosphorylation, and VEGFR2) were further investigated in an independent cohort of 750 PitNET cases.

PitNET is the most common neuroendocrine tumor and one of the most common intracranial tumors, leading to severe clinical manifestations.^3^ While treatments include surgery, radiotherapy and medication, the management of frequently recurrent aggressive PitNET (i.e., refractory PitNET) remains clinically challenging.^4^ According to the WHO 2017 classification, PitNETs can be categorized into ten subtypes based on IHC of TF markers and hormone expression. This classification also indicates several tumors with a higher probability of recurrence than typical PitNETs, including tumors with elevated proliferative activity and the special variants of adenomas.^5^ However, guidance for selecting a treatment strategy using this classification system is limited.^56^ To explore new potential treatments for PitNET, we conducted a proteogenomic study to identify innovative drug targets, which resulted in reclassification of PitNETs into seven clusters based on TF expression and molecular characteristics in multi-omics data (Fig. 6a). Each cluster has specific potential treatment targets, and this new, clinical treatment-oriented classification represents a major breakthrough for selecting appropriate therapeutic interventions for this highly heterogeneous disease.

PDL1 has been widely studied in many other tumors, although systematic evaluation of PD1/PDL1 therapy has not been conducted in PitNET. Previous works have included IHC staining of PDL1^57^ and case reports of immunotherapy for PitNET.^58^ Here, PDL1 expression and immune status were investigated for all PitNET subtypes using the combined multi-omics data. Considering the severe side effects of anti-PDL1 therapy (e.g., neuromuscular disorders, myocarditis and intraocular inflammation),^59^ it is necessary to select patients who are sensitive to and could benefit from this treatment. Here we identified two PitNET clusters (GH^enrich^ and TSH/silent PIT1^enrich^) which exhibited high PDL1 expression in our proteogenomics data. Interestingly, immune cell subtyping analysis revealed that high PDL1 expression was significantly associated with JAK1/STAT1 activation in the Immune-exhausted cluster. Other studies have shown that therapies targeting STAT1 can be combined with anti-PDL1 antibody for patients resistant to immune checkpoint blockade,^60^ leading us to propose that JAK1/STAT1 inhibitor plus anti-PDL1 antibody could be potentially used to treat these PitNET patients. This possibility suggests another major implication of our findings for the application of immune therapies to treat a subset of PitNET clusters.

In total, we identified five transcriptomic clusters, seven proteomic clusters, and seven phosphoproteomic clusters in the 200 PitNET cohort. The vast majority of clusters identified in TPIT lineage, SF1 lineage, and NULL tumors were recapitulated in each of the multi-omics datasets. We focused on proteomic classification since these data could distinguish EMT^PRO^, TSH/silent PIT1^enrich^, PRL^enrich^, and GH^enrich^ clusters within the PIT1 lineage, thus better reflecting clinicopathological subtypes than either the transcriptomic or phosphoproteomic datasets. We then examined the most likely treatment targets of each cluster. The targets of available drugs for PitNET, such as DRD2 and SSTR2/5, were confirmed in corresponding clusters, suggesting the potential effectiveness of dopamine agonist and somatostatin analogs in these clusters. For the other treatment targets, although some of them were previously identified in other cancers,^41,61^ the large majority are described here for the first time in PitNETs, such as EMT, EGFR T693 phosphorylation, CDK6 and PDL1.

By systematic, combined analysis of genomic, transcriptomic, and proteomics datasets, we identified the *cis* effects of chromosome 20q that lead to cell cycle upregulation in the invasive PIT1 lineage, which is consistent with another previous report.^19^ Further investigation of chromosomal instability indicated that GNAS protein overexpression was likely due to the occurrence of *GNAS* copy number gain. Moreover, *GNAS* copy number gain and *GNAS* mutation were mutually exclusive. *GNAS* mutation has been well studied and is uniquely present in GH-secreting PitNETs with characteristically lower capacity for invasion and higher serum levels of GH.^62^ Interestingly, PIT1 lineage patients with *GNAS* copy number gain did not exclusively harbor GH-secreting PitNETs, and may present with distinct clinical features, such as highly invasive tumors, in sharp contrast with a report by Hage et al., who found no differences correlated with changes in 20q chromosome copy number, where *GNAS* is located.^28^ Further studies are required to verify these findings and to identify the differences between *GNAS* mutation and *GNAS* copy number gain in their respective mechanisms driving PitNET phenotype.

The proteogenomics datasets generated in this study enabled the identification of seven clusters and establishment of a biologically, prognostically, and therapeutically relevant classification system for PitNETs. Notably, EMT^PRO^ cluster presents a highly invasive malignancy characterized by high expression of EMT-related proteins, which is consistent with a previous report that showed EMT marker expression is correlated with tumor diameter and invasion in PitNET.^63^ The potential therapeutic value of EMT suppression for treating patients in the EMT^PRO^ cluster warrants attention. Alternatively, immune therapy may be an effective treatment strategy in these patients, since these tumors also feature immune infiltration. Immunosuppressive mediators such as CTLA4 are overexpressed in the EMT^PRO^ cluster, suggesting that immunotherapies may improve outcomes for these patients.

Another invasive tumor cluster is silent TPIT^enrich^, which consists of the non-hormone secreting TPIT lineage, and is characterized by EGFR T693 phosphorylation in our datasets. A recent report also linked EGFR T693 phosphorylation in silent PitNETs with worse prognosis.^64^ EGFR was previously identified as a druggable target in ACTH PitNETs, another TPIT lineage subtype that exhibits ACTH hypersecretion.^11^ In the present study, we found that EGFR is overexpressed in all TPIT lineage tumors and further discovered three distinct modes of EGFR signaling pathway activation that led to dysregulation of different downstream pathways and opposite clinical features. More specially, *USP8* mutation or EGFR ligand activation in ACTH PitNETs are associated with decreased tumor diameter and ACTH secretion, as previously reported.^16^ By contrast, EGFR T693 phosphorylation in silent TPIT activates the PI3K-AKT-mTOR pathway and results in invasive tumors without hormone hypersecretion. This discrepancy suggests that different target therapies may be effective depending on activation mode for TPIT patients: for ACTH patients with *USP8* mutation, inhibiting USP8 and/or EGFR activity may be an effective therapeutic approach; for ACTH_*USP8* WT patients whose EGFR ligands are activated, EGFR monoclonal antibody (mAb) might effectively prevent ligand-induced pathway activation; finally, for silent TPIT patients, in which EGFR T693 is highly phosphorylated, EGFR tyrosine kinase inhibitors (TKIs) may provide a good response.

NULL PitNETs are more aggressive than other clinicopathological subtypes,^65,66^ and no therapeutic agents have yet shown efficacy in patients with these tumors. In the present study, we indicate that SF1 lineage and NULL tumors have similar expression patterns in transcriptomics, proteomics, and phosphoproteomics datasets. Based on this similarity between subtypes evident in the multi-omics data, we suggest that the NULL and SF1 lineage subtypes can be functionally combined into a single SF1/NULL^enrich^ cluster. However, in clinic, other factors should be considered. For example, Tebani et al. identified a NULL case showing high TPIT expression with moderate ACTH expression levels.^67^ In addition, metastases of neuroendocrine tumors from other primary locations should be considered in the differential diagnosis of so-called NULL tumors.

In our study, the SF1/NULL^enrich^ cluster showed characteristic upregulation of glycolysis and citrate cycle. Analyses of transcriptomics and proteomics datasets also led to the identification of several highly upregulated mitochondrial citrate cycle enzymes, including IDH1, IDH2, IDH3A, IDH3B and IDH3G (Supplementary information, Fig. S7a). Among them, Tebani et al. recently reported that IDH1 expression in SF1 was higher than that in TPIT and PIT1 tumors,^67^ which is consistent with our findings. Further analysis revealed that VEGF signaling, activated by VEGFR2 and VEGFA, was obviously aberrantly upregulated in the SF1/NULL^enrich^ cluster, suggesting that antiangiogenic therapies (e.g., apatinib) and VEGFA-targeting fluorescence-assisted surgery could potentially improve outcomes for these patients.^68,69^

This integrative proteogenomic analysis substantially expands the current understanding of PitNET pathophysiology and can guide the development and selection of effective treatment strategies.

## Materials and methods

### Specimen acquisition

#### Patient recruitments

Two cohorts of patients were recruited: one for genomics, transcriptomics, proteomics, and phosphoproteomics analysis (200 PitNETs), the other for IHC validation (750 PitNETs). All underwent surgeries at the Department of Neurosurgery at Huashan Hospital, an affiliate of Shanghai Medical College, Fudan University. Patients in the 200 PitNET cohort underwent surgeries between 2018 and 2020, while the 750 PitNET cohort was collected between 2010 and 2014. Both cohorts only included subjects without previous malignancies. Clinical data and follow-up information of all patients were collected from medical records retrospectively and were shown in our study (Supplementary information, Tables S1 and S9). Seven anterior pituitary gland tissues were obtained from donors without evidence of any endocrine disease. All patients have signed written informed consent, and the ethics committee at Huashan Hospital has approved the study (KY2021-498). The study was performed in accordance with the Declaration of Helsinki.

The diagnosis of PitNET was based on clinical manifestations, imaging, endocrine laboratory tests, and post-operative IHC staining according to previously described criteria and currently accepted standard guideline.^9,70–74^ The following IHC staining was performed to classify clinicopathological subtypes: TFs including PIT1, TPIT, and SF1, pituitary hormones including PRL, GH, TSH, FSH, LH, and ACTH, along with cytokeratin 8 (CAM5.2), Syn, Ki67 (MIB1), and ER (estrogen receptor). After surgical resection, histological diagnosis was confirmed in a blinded fashion by at least two senior pathologists.

PitNET invasion was assessed on two dimensions: (1) Radiology invasion: based on pre-operative MRI imaging, PitNETs classified as Hardy’s modified classification grades III, IV and/or stages C, D and E, or Knosp classification grades III and IV are considered invasive;^75,76^ (2) Surgery invasion: invasion of the dura, cavernous sinus, mucosa or bone in sphenoidal sinus based on intra-operative findings and pathological examination.

#### Tumor sample collection and characterization

Tumor fragments were removed and collected by experienced neurosurgeons (Yao Zhao and Yongfei Wang) during surgery. To avoid any contamination by normal APG tissue or para-tumoral connective tissue, the most representative tumor specimens with minimal hemorrhage and necrosis were carefully picked from the middle of the tumor, which were then rapidly frozen in liquid nitrogen within 15 min and subsequently stored at ‒80 °C ultra-low temperature freezers.

The average weight of a tumor tissue was 55 mg. Acceptable PitNET tissue segments had to contain more than 80% tumor cell nuclei and less than 20% tumor necrosis at the top and bottom of histological sections decided by two pathologists. To facilitate the homogeneity of samples, each tissue was homogenized via cryopulverization and then aliquoted for subsequent DNA (~5 mg), RNA (~5 mg) and protein (~45 mg) extraction from the same tissue sample. In addition, paired perioperative blood was also collected for WES to call somatic mutation of each PitNET.

The 200 PitNET cohort was adopted for integrated proteogenomic analyses, including paired WES (*n* = 200), RNA-seq (*n* = 194), proteomics profiling (*n* = 200), and phosphoproteomics profiling (*n* = 194). The 750 PitNET cohort was used to construct tissue microarray for large-scale IHC staining, including: GNAS, ZEB2, TWIST1, PDL1, VEGFR2, EGFR, and EGFR T693.

### WES

#### DNA extraction and DNA quantification

Total DNA was extracted from approximately 5 mg cryopulverized PitNET tissues using QIAamp DNA Mini Kit (Qiagen-51306) according to the manufacturer’s instructions. The total DNA of blood was extracted from the 1 mL blood using QIAamp DNA Blood Mini Kit (Qiagen-51106) according to the manufacturer’s instructions. Then the concentration and integrity of the total DNA were detected by Qubit 2.0 fluorometer dsDNA HS Assay (Thermo Fisher Scientific) and agarose electrophoresis. OD_260_/OD_280_ was measured by NanoDrop2000 (Thermo Fisher Scientific). About 300 ng high-quality DNA (OD_260_/OD_280_ = 1.8‒2.0) of each PitNET tissue was adopted subsequently to construct the sequencing library.

#### WES library preparation

The 300 ng genomic DNA samples were sheared with Covaris LE220 Sonicator (Covaris) to obtain an average size of 150‒200 bp. DNA libraries were constructed using SureselectXT reagent kit (Agilent) according to the manufacturer’s instructions. End repair mix (component of SureselectXT) was used for repairing the 3’ and 5’overhangs of the fragments, which were subsequently purified with Agencourt AMPure XP Beads (Beckman). A tailing Mix (component of SureSelectXT) was used for adding ‘A’ tail to the purified fragments, which were then ligated to an adapter using DNA ligase, a component of SureselectXT. Herculase II Fusion DNA Polymerase (Agilent) was used for the adapter-ligated DNA fragments amplification. Finally, the SureSelect Human All Exon V5kit (Agilent) was used to pre-capture libraries containing exome sequences.

#### Illumina sequencing

The Qubit 3.0 fluorometer dsDNA HS Assay (Thermo Fisher Scientific) was used to determine the DNA concentrations of the enriched sequencing libraries. Agilent BioAnalyzer 4200 (Agilent) was used to analyze the size distribution of the resulting sequencing libraries. Paired-end sequencing is performed using an Illumina NovaSeq6000 system according to Illumina’s 2× 150 paired-end sequencing protocol. WES was conducted with a mean coverage depth of 297× (range: 250‒412×) for tumor samples and 156× (range: 120‒208×) for paired blood samples, consistent with the recommendations for WES.^77,78^

### WES data analysis

#### Quality control

The first step in our data analysis was quality control to ensure that raw data had good metrics and no significant biases which may affect the following analysis. In this study, read quality was evaluated for all samples by using FastQC (v0.11.9) software with the default parameters.

#### Filter of raw data

The adapter sequence of raw data (Fastq) was removed using Fastp (v0.22.0). Then, the reads with more than 75 bp were preserved (named clean reads) and used for downstream analysis

#### Sequence alignment

Clean reads were mapped to the human (*Homo sapiens*) genome (version hg19) using the BWA-MEM (v0.7.17) algorithm to generate the bam file, followed by marking the PCR reads in the bam, rearranging the regions that may include Indel mutations, and recalibrating the quality of each base pair.

#### Variant detection and filtering

Gene variants are base changes that occur in the genome, such as point mutations caused by single base changes, or insertions, deletions, and duplications of multiple bases. In this study, the pipeline of Sentieon software (20201001) was applied to detect InDel (small insertions and deletions) and SNV (Single Nucleotide Variants). The variants were then annotated by Annovar (Date 20180416).^79^ Databases of the 1000 Genomes^80^ and Exome Aggregation Consortium (ExAC) were used to filter the germline variants. High-quality somatic variants were obtained through a stringent downstream filter incorporating the following criteria: strand bias ratio ≥ 0.1; Variant Allele Fraction (VAF) ratio < 0.2; variant supporting reads ≥ 5; ExAc_EAS ≤ 1%; Subsequently, mutations located in the non-coding regions were eliminated. A total of 12,782 non-silent somatic SNV and indel calls for tumor samples were obtained in contrast to matched blood samples (200 pairs). In our study, the transcriptional version of the *GNAS* variants is NM_001077489 and that of the *USP8* variants is NM_001128610.

#### CNA analysis

CNVkit software can analyze the CNA of single sample and paired tumor samples. In this project, CNAs were determined using CNVkit version 0.99. And a 200 bp bin size was used to analyze the whole-genome CNA. Segment files of every 1000 were input in GISTIC2 to determine significantly amplified or deleted regions across all PitNET samples. Thresholds with the following parameters were used to exclude false positives: -brlen 0.5 -broad 1 -conf 0.9. To identify the genes with CNAs, the correlation between copy numbers and the abundances of mRNA, protein, or phosphoprotein were tested by Spearman correlation coefficients. Genes displaying significant correlations with mRNAs, proteins, and phosphoproteins with adjusted *P* values smaller than 0.05 were selected. Correlations were visualized using multiOmicsViz (R package).

### RNA-seq

#### RNA extraction, library preparation, and Illumina sequencing

Approximately 5 mg cryopulverized PitNET tissues or APGs were preserved to extract total RNA using TRIzol® Reagent (Invitrogen) and RNeasy minElute spin column (Qiagen). Then 2100 Bioanalyser (Agilent) and NanoDrop (Thermo Fisher Scientific) were used to quantify the integrity of the total RNA. About 500 ng high quality RNA sample was obtained to construct sequencing library.

Sequencing libraries were generated using Ribo-off® rRNA Depletion Kit (H/M/R) (Vazyme #N406) and VAHTS® Universal V6 RNA-seq Library Prep Kit for Illumina (#N401-NR604), followed by adding index codes to attribute sequences to each sample. The libraries were sequenced on an Illumina platform and 150 bp paired-end reads were generated.

### RNA-seq data analysis

#### Quality control

The first step in our data analysis was quality control to ensure that raw data had good metrics and no significant biases which may affect the following analysis. In this study, FastQC (v0.11.9) with default parameters was applied to calculate read quality for all PitNET samples.

#### Filter of raw data

The adapter sequence of raw data (Fastq) was removed using Fastp (v0.22.0). Then, the reads with more than 75 bp were preserved (named clean reads) and used for downstream analysis

#### Sequence alignment

Then the clean reads of each PitNET sample were mapped to the human (*Homo sapiens*) genome (version hg19) by using STAR (v2.4.2a) software with default parameters and were annotated with transcriptome database (gencode v19).

#### Gene expression estimation

Expression estimation of gene and transcript was performed by using RSEM (V1.2.29) with setting-estimate-rspd parameter to estimate the distribution of the starting position of the sequencing sequence (RSPD) with other parameters in default. The relative abundance of the transcript was quantified based on normalized metric named FPKM. Transcripts whose FPKM score > 1 were preserved.

### Peptide preparation for MS analysis

#### Protein extraction and tryptic digestion

As for protein extraction and tryptic digestion, approximately 45 mg cryopulverized PitNET tissues or APGs were homogenized separately in an appropriate volume of Urea lysis buffer (8 M urea, 100 mM Tris hydrochloride, pH 8.0) containing protease and phosphatase inhibitors (Thermo Scientific). The lysate was centrifuged at 4 °C 16000× *g* for 15 min for clarification, and the BCA method was applied to measure the protein concentration. About 4900 μg protein was extracted from each sample. Next, protein samples were replenished with a final concentration of 5 mM dithiothreitol (DTT) before incubation for 30 min at 56 °C, which were then supplemented with a final concentration of 20 mM iodoacetamide (IAA), and finally were incubated at room temperature in the dark, according to the FASP procedure.^81^ After 30 min incubation, samples were supplemented with DTT to a final concentration of 5 mM and maintained for another 15 min in the dark. Protein samples were centrifuged at 12,000×  *g* for 20 min in 30 kD Microcon filtration devices, and then were washed twice with Urea lysis buffer and 50 mM NH_4_HCO_3_. Protein samples were then digested with trypsin at 37 °C overnight with an enzyme to protein mass ratio of 1:25. Peptides were dried with SpeedVac (Eppendorf).

#### Phosphopeptide enrichment

The peptide concentration was determined using a NanoDrop 2000C spectrophotometer (at 280 nm). Approximately 300 μg peptides were then enriched with the High-Select™ Fe-NTA Phosphopeptide Enrichment Kit (Thermo Fisher Scientific, A32992), following the manufacturer’s recommendations.

#### Liquid chromatography-tandem MS

Digested peptides were analyzed on an Easy-nLC 1200 nanoflow LC system tandem with a Fusion Lumos (Thermo Fisher Scientific). Peptide samples were loaded into a homemade trap column (100 μm × 2 cm; pore size, 120 Å; particle size, 3 μm; SunChrom; USA), and then separated with a gradient of 4%–100% mobile phase B (80% acetonitrile and 0.1% formic acid) at a flow rate of 600 nL/min for 150 min by a homemade silica microcolumn (150 μm× 30 cm; pore size, 120 Å; particle size, 1.9 μm; SunChrom; USA).

LC-MS/MS based proteomic and phosphoproteomic experiments were conducted with Field Asymmetric Ion Mobility Spectrometry (FAIMS). FAIMS voltages were set to ‒40 V, ‒60 V and ‒80 V, respectively, and other parameters were consistent and set as follows: protein quantification consisting of an MS1 scan at a resolution of 120,000 (at 200 m/z) with an AGC value of 5e5, max injection time of 50 ms and scan range 350–1500 m/z, MS2 scans with higher-energy collision dissociation detected in the Ion Trap first (Ion trap scan rate = rapid, isolation window 1.6 m/z, max injection time 10 ms, AGC target 1e4, normalized collision energy of 30%). The dynamic exclusion time of previously obtained precursor ions was 45 s, cycle time = 1 s.

### MS data analysis

#### Identification of peptide and protein

MS raw files of proteomics data were processed with “Firmiana”, a one-stop proteomic cloud platform^82^ against the human RefSeq protein database (updated on 04-07-2013) in the National Center for Biotechnology Information using the Mascot 2.4 search engine. A mass tolerance of 20 ppm for precursor and 0.5 Da for production were allowed. Up to two missed cleavages were allowed. Carbamidomethyl (C) was set as fixed modification, and N-acetylation and oxidation of methionine were set as variable modifications. To control the quality of protein identification, a target-decoy-based strategy was employed to control the FDR of both the peptides and proteins to less than 1%. Percolator was used to obtain the probability value (q value), and to validate the FDR (measured by the decoy hits) of every peptide-spectrum match (PSM) lower than 1%. Thereafter, peptides shorter than seven amino acids were removed. For peptide identification, the cutoff ion score was set as 20. To obtain more stringent quality control, all PSMs in all fractions were combined for protein quality control. The q values of both the target and decoy peptide sequences were dynamically increased until the corresponding protein FDR was less than 1% using the parsimony principle. Finally, proteins with at least two unique peptides were selected for further investigation to reduce the false positive rate.

MS raw files of phosphoproteomics data were searched against the human refseq protein database (updated on version 04/07/2013, 27,414 proteins) with Proteome Discoverer (version 2.3.0.523) using Mascot^83^ (version 2.3.01) engine with a percolator.^84^ Oxidized methionine, N-term acetylation, and phospho (STY) were set as variable modifications, and carbamidomethyl cysteine was used as a fixed modification. The FDR of peptide and protein was set at 1%. The tolerance for spectra search allowed 20 ppm mass tolerance for the precursor. Up to two missed cleavages were allowed.

#### MS quantification of proteins and phosphoproteins

Proteomics datasets were quantified using Firmiana, and both the results and raw data from the mzXML file were loaded. Then, each identified peptide was retrieved according to the identification information of MS1 to obtain the extracted-ion chromatogram (XIC), and the abundance was estimated by calculating the area under the extracted XIC curve. Non-redundant peptide list was used to assemble the proteins according to the parsimony principle. Protein abundance was then estimated using a traditional label-free, intensity-based absolute quantification (iBAQ) algorithm, which divided the protein abundance (derived from intensities of the identified peptides) by the number of theoretically observable peptides. Fraction of total (FOT) is a relative quantification value defined as a protein’s iBAQ divided by the sum of the iBAQ of all proteins identified in an experiment, calculated as the normalized abundance of a particular protein in the experiment. Finally, for the ease of presentation, the FOT was further multiplied by 1e5.

Phosphoproteomics datasets were quantified using the Proteome Discover (version 2.3). For the phosphoprotein abundance calculation, the non-redundant phosphopeptide list was used to assemble the proteins according to the parsimony principle. For phosphosite localization, phosphosite confidence was determined using ptmRS^85^ and a phosphosite probability > 0.75 was used for further analysis. The phosphoprotein abundance was calculated from the sum of phosphopeptide abundance.

#### Missing value imputation

Proteins and phosphosites with a missing rate < 50% were imputed separately on the data from clinicopathological subtype, which is consistent with previous report.^24^ The missing values were imputed by K-nearest neighbor (KNN) algorithm using the 5 nearest neighbors based on “impute” R package (10.18129/B9.bioc.impute).

#### Batch effect analysis

Dip statistic test and PCA implemented in R v.4.0.2 were adopted to evaluate the batch effects in our study with regard to the following two variables: sample type and batch identity. The density plots of the the mRNAs, proteins and phosphosites have an expected unimodal distribution by dip statistic test, indicating that the samples passed the quality control (Supplementary information, Fig. S1c). In the PCA procedure, the results also displayed that batch effects were negligible for batch identity but were significant for the clinicopathological subtypes (Fig. 1h; Supplementary information, Fig. S1e, f).

#### Quality control of the MS data

Digested peptides of HEK293T cell (National Infrastructure Cell Line Resource) were acquired with LC-MS/MS to evaluate the stability of instrument every three days. The HEK293T cell was digested and analyzed using the same protocol and conditions as PitNET samples. The Spearman’s correlation coefficient was computed for all quality-control runs in R v.4.0.2, and the results are displayed in our study (Supplementary information, Fig. S1d). The average correlation coefficient among the 23 HEK293T cells was 0.91, rangeing from 0.87 to 0.95.

### Global proteomics analysis

#### Differential protein analysis

Wilcoxon rank-sum test was applied to calculate the differentially expressed proteins between subtypes. Up or down-regulated proteins in a specific subtype were identified as differentially expressed proteins compared with other subtype samples (ratio > 2, Wilcoxon rank-sum test, Benjamini-Hochberg adjusted *P* < 0.05).

#### Pathway enrichment analysis

Gene sets of molecular pathways from the KEGG^86^/Hallmark^87^/Reactome^88^ databases were applied to compute pathways. Differentially expressed proteins defined in different clinicopathological subtypes or omic clusters were subjected to pathway enrichment analysis in ConsensusPathDB (http://cpdb.molgen.mpg.de/) with FDR < 0.05.

### Phosphoproteomics analysis

#### Differential phosphoprotein and phosphopeptide analysis

Wilcoxon rank-sum test was used on PitNET samples to identify differential abundance of phosphoproteins and phosphosites between subtypes. Upregulated or downregulated phosphoproteins and phosphosites in tumor samples were defined as differentially expressed phosphoproteins and phosphopeptides in each specific subtype and were defined as differentially expressed proteins compared with other subtype samples (ratio > 2, Wilcoxon rank-sum test, Benjamini-Hochberg adjusted *P* < 0.05).

### Multi-omics data analysis

#### Analysis of significantly mutated genes

MutSigCV (https://software.broadinstitute.org/cancer/cga/mutsig, version 1.4) and OncodriveCLUST were used for identifying significantly mutated genes with default parameters. The Benjamini and Hochberg method was adopted to convert the final *P* values to q values.^89^ Significant mutations were determined in genes with q ≤ 0.1.

#### Co-occurrence and mutual exclusivity analysis of mutations

In our mutational dataset, Fisher’s exact test was used to investigate co-occurrence and mutually exclusive mutated genes.

#### Mutation signature analysis and TMB

Mutational Signatures in Cancer (MuSiCa) software^90^ was used to jointly infer mutational signatures in 200 PitNET tumors. The 96 mutation vectors (or contexts) generated from somatic SNVs based on six base substitutions (C > A, C > G, C > T, T > A, T > C, and T > G) within 16 possible combinations of neighboring bases for each substitution were used as input data in order to infer their contributions to the observed mutations. To infer their exposure contributions, non-negative matrix factorization (NMF) approach was applied in MuSiCa to decipher the 96 × 159 (i.e., mutational context-by-sample) matrix of 30 known COSMIC cancer signatures (https://cancer.sanger.ac.uk/cosmic/signatures). The number of somatic mutations (including base substitutions and indels) in the coding region was defined as TMB. To compute TMB for each PitNET patient, the total number of mutations calculated was divided by the size of the coding sequence region of the Agilent SureSelect Human All Exon V6.

#### Effects of CNAs

Somatic CNAs affecting the expression levels of mRNA, protein, and phosphoprotein in either “*cis*” (within the same aberrant locus) or “*trans*” (remote locus) mode were calculated using multiOmicsViz (R package). Spearman’s correlation analysis (FDR < 0.05) was performed for CNA-mRNA correlation, CNA-protein correlation and CNA-phosphoprotein correlation, consistent with the same FDR cutoff value in recent studies.^22,42^ The CAGs used in Fig. 2e were from CAGs defined by Bailey et al.,^25^ Mertins et al.^91^ and Vogelstein et al.^92^

#### Evaluation of mRNA-protein correlation

A total of 6115 genes that correspond to mRNA and protein abundances were used to evaluate gene-wise mRNA-protein correlation. Spearman correlation coefficient between paired mRNA expression and protein abundance was measured. The *P* values of the correlation coefficient were calculated and adjusted by the FDR correction. As a result, the median Spearman correlation coefficient of matched genes is 0.24. To identify cellular pathways with the largest and smallest mRNA-protein correlations, GSEA^93^ was performed based on correlation-ranked list of genes.

#### PCA

We performed PCA on 200 PitNET tumor samples and 7 APGs to illustrate the omic difference between each subtype/cluster samples. To visualize representation of omics separation of samples, we used the R package factoextra.^94^ The 95% confidence coverage was represented by a colored ellipse for each clinicopathological subtypes, which was calculated from the mean and covariance of points in each particular group.

#### Consensus clustering analysis for proteome, transcriptome, and phosphoproteome

Consensus clustering was performed to identify proteomic clusters of PitNETs using the ConsensusClusterPlus and CancerSubtypes package in R.^31,95^ We selected the top 1150 proteins from the proteins expressed in at least 50% of the samples for clustering across 200 samples. Parameters were reps=1,000, pFeature = 0.8, pItem = 0.8, clusterAlg = “hc”, distance = “spearman” in the range of 2 to 10 clusters. The consensus matrices of k = 4, 5, 6, 7, and 8 clusters are shown in our study (Supplementary information, Fig. S4a). The delta plot of the relative change in the area under the cumulative distribution function (CDF) curve, and the average silhouette distance for consensus clusters were calculated to estimate the average pairwise consensus matrix within consensus clusters (Supplementary information, Fig. S4a). We then determined the consensus matrix of k = 7 as the best solution for clustering as it presents the most separated clusters compared with other k value. Furthermore, compared to k = 7, when using k = 8, the consensus matrix was also almost clearly divided into seven consensus clusters rather than eight. In addition, the largest average silhouette width (0.81) for k = 7 suggested the highest similarity of samples in each cluster allocated by it.

To compare proteomic clusters with other omic datasets, transcriptomic and phosphoproteomic clusters were identified using a similar procedure. For the clustering of transcriptomics data, 2735 mRNAs among the top 20% most varied mRNAs were selected in 194 tumor samples (Supplementary information, Fig. S4b). For the clustering of phosphoproteomics data, 1244 phosphoproteins among the top 50% most varied phosphoproteins were selected in 194 tumor samples (Supplementary information, Fig. S4c). Parameters were reps = 1,000, pItem = 0.8, pFeature = 0.8, clusterAlg = “pam”, distance = “spearman” in the range of 2 to 10 clusters. In addition to considering the factors used in proteomic clusters, patterns of concordance across data types and with histological diagnosis were considered. We finally selected 5 transcriptomic clusters and 7 phosphoproteomic clusters for further analysis.

#### GSEA

GSEA was performed by the GSEA software (https://www.gsea-msigdb.org/gsea/index.jsp).^93^ Gene sets including KEGG, Reactome, Gene Ontology and HALLMARK downloaded from the MsigDB (v7.4, https://data.broadinstitute.org/gsea-msigdb/msigdb/release/7.4/) were set as background.

#### Immune subtype identification

Immune score, stromal score and tumor purity were inferred using the R package ESTIMATE v1.0.11^37^ using transcriptome data (Supplementary information, Table S7). The abundance of 64 cell types in 194 samples were estimated using xCell (https://xcell.ucsf.edu/)^47^ based on transcriptomic profiles. Cell types which were detected in at least 10% of the patients were used for further consensus clustering, using the R packages ConsensusClusterPlus^31^ (clusterAlg = “pam”, distance = “spearman”). (Fig. 5a; Supplementary information, Table S7).

#### Pathway ssGSEA

To better understand biological processes and pathway scores for each sample at the protein and mRNA levels, including 200 PitNET tumor samples and 7 APGs, we applied ssGSEA^96^ using the GSVA package.^97^ For this analysis, gene sets (KEGG, Reactome, Gene Ontology and HALLMARK) downloaded from the MsigDB (v7.4, https://data.broadinstitute.org/gsea-msigdb/msigdb/release/7.4/) were set as background.

#### Kinase activity inference

The phosphoproteomics data were processed using NetworKIN^98^ to predict kinases for every identified phosphosite. Known substrates from PhosphoSitePlus^99^ and UniProt used in Kinase Activity Inference were used to generate substrate sets, which were further predicted from NetworKIN with a NetworKIN score ≥ 5. The similar approach of predicting kinase activity was also used in Clinical Proteomic Tumor Analysis Consortium (CPTAC) works.^24,41^ Kinase Activity Inference was further evaluated from kinase-substrate pairs using ssGSEA^96^ via the GSVA package.^97^

#### TF activity inference

TF activities for 200 PitNET tumors were computed using ssGSEA^96^ via the GSVA package.^97^ TF targets obtained from DoRothEA (v1.6.0)^100^ were set as background.

#### MGPS

The MGPS was computed based on gene expression data of genes contained in the proliferation signature from Ellis et al.^101^ ssGSEA score of the package GSVA^97^ was used for MGPS calculation. The similar approach of calculating MGPS was also employed in CPTAC works.^41,61,102^

#### PROGENy scores

PROGENy^46^ was used to generate activity scores for EGFR, VEGF, Hypoxia, etc. based on RNA expression data. Tumor RNA expression values were submitted to PROGENy.

#### Survival analysis

The coefficient value was calculated from Cox proportional hazards regression analysis. Values with *P* < 0.05 were chosen for Cox regression multivariate analysis. Kaplan-Meier survival curves (log-rank test) were used for progression-free survival (PFS) of the patients. Survminer (version 0.2.4, R package) with maximally selected rank statistics (http://r-addict.com/2016/11/21/Optimal-Cutpoint-maxstat.html) was used to determine the optimal cut-off point of a given protein, phosphoprotein, or phosphosite for the following calculation including Kaplan-Meier analysis, log-rank test.^103^

### Immunohistochemistry and image analysis

#### Antibodies

**Table:** 

Antibodies	Source	Identifier
PDL1 (E1L3N)	Cell Signaling Technologies	Cat# 13684, RRID: AB_2687655
CDK6 [EPR4515]	Abcam	Cat# ab124821, RRID: AB_10999714
EGFR [EP38Y]	Abcam	Cat# ab52894, RRID: AB_869579
EGFR T693	Immunoway	Cat# YP0087
GNAS [EPR24177-24]	Abcam	Cat# ab283266
VEGF Receptor 2 [55B11]	Cell Signaling Technologies	Cat# 2479, RRID: AB_2212507
TWIST1	ABclonal	Cat# A15596, RRID: AB_2763001
ZEB2 (Clone 1E12)	Origene	Cat# TA802113, RRID: AB_2616296
Pan-Keratin (C11)	Cell Signaling Technologies	Cat# 4545, RRID: AB_490860
Fibronectin/FN1 (E5H6X)	Cell Signaling Technologies	Cat# 26836

#### Staining of tissue sections

Patient tumor samples were fixed in 4% paraformaldehyde for 24 h, dehydrated by gradient ethanol and xylenes, then embedded in paraffin. Paraffin blocks were cut into 3 μm sections for IHC staining, IF staining, or HE staining. For IHC, slides were deparaffinized and rehydrated through xylenes and graded ethanol, followed by antigen retrieval using heat-induced epitope retrieval (HIER). After blocking endogenous peroxidase and nonspecific binding sites (0.3% H_2_O_2_ and 5% normal goat serum, respectively), primary antibodies were applied at 4 °C overnight. Slides were incubated with Dako REAL™ EnVision™ HRP rabbit/mouse (belong to K5007, DAKO, Glostrup, Denmark) at room temperature for 20 min, followed by applying Dako REAL™ DAB + CHROMOGEN and Dako REAL™ substrate buffer (belonging to K5007, DAKO, Glostrup, Denmark) to visualize staining signals under light microscopy, finally counterstained by hematoxylin solution. Stained slides were scanned by Ocus (Grundium, Tampere, Finland) and analyzed with Qupath software (see below). For IF staining, procedures before primary antibodies incubation were the same as IHC, except for H_2_O_2_ blocking. Slides were incubated with primary antibodys at 4 °C overnight, followed by incubation with Goat Anti-Rabbit Alexa Flour 568 (1:500, ab175695, Abcam) and Goat Anti-Mouse Alexa Flour 488 (1:500, 115-545-003, Jackson ImmunoResearch). Finally, the slides were counterstained with DAPI (1:1000, HY-D0814, MCE) and mounted in an antifade solution (Fluoromount-G, 0100-01, SouthernBiotech).

#### Image analysis

HE and IHC images were scanned by Ocus whole-slide scanner (Grundium, Tampere, Finland) and processed with Qupath software 0.3.0.^104,105^ For IHC, images are preprocessed by the built-in stain vector estimator. Cells with shape and stain parameters in each area were identified by built-in cell detection function via nucleus stain (hematoxylin). For each antibody, the mean DAB optical density (OD) thresholds for positivity grades were decided according to the staining pattern and intensities, and then were applied uniformly to all samples. The H-score was calculated as the percentage of tumor cells with positive staining multiplied by the average intensity (0‒3) of positive staining for PDL1, CDK6, EGFR, EGFR_T693, GNAS, TWIST1, and ZEB2. Besides, the positive locations of VEGFR2 were mainly located at elongated endothelial cells, which cannot be identified cell by cell in Qupath. The quantitative results of VEGFR2 were calculated as positive area divided by total tissue area (positive area ratio) for each sample. For HE, we built a machine learning pixel classifier within Qupath, which learns typical tumor cell-enriched and stroma cell-enriched areas and gives classifications of either tumor or stroma region in each sample. Scripts of the whole-slide images analysis protocol above were created, batch performed on each set of images and further checked by two expert pathologists. All quantifications were evaluated blind to patient clinical characteristics.

### Outlier process in H-score analysis

Winsorization^106^ is used to limit extreme values in the H-score data to reduce the effect of possibly spurious outliers. The H-score below the boundaries of the 5th percentile was set to the 5th percentile, and the H-scores above the boundaries of the 95th percentile was set to the 95th percentile.

### Statistical analysis

Standard statistical tests, including but not limited to Chi-square test, Fisher’s exact test, Wilcoxon rank-sum test, Kruskal-Wallis test, and Log-rank test, were adopted to analyze the clinical data. For categorical variables vs categorical variables, Fisher’s exact test was used in a 2 × 2 table; otherwise, the Chi-square test was used. For categorical variables vs continuous variables (e.g., pathway scores, kinase activity scores and TF activity scores), the Wilcoxon rank-sum test and Kruskal-Wallis test were used to test whether any of the differences among the subgroups were statistically significant; and Spearman correlation was used for continuous variables vs continuous variables. Statistical significance was considered at a *P* value < 0.05. To account for multiple testing, the *P* values were adjusted using the Benjamini-Hochberg FDR correction and was considered significant when < 0.05. Kaplan-Meier plots (log-rank test) were used to describe PFS. All analyses of clinical data were performed in R (version 4.0.2).

## Supplementary information

Fig. S1

Fig. S2

Fig. S3

Fig. S4

Fig. S5

Fig. S6

Fig. S7

Fig. S8

Fig. S9

Table S1

Table S2

Table S3

Table S4

Table S5

Table S6

Table S7

Table S8

Table S9

## Data Availability

The MS proteomics and phosphoproteomics raw data have been deposited to the ProteomeXchange Consortium (http://proteomecentral.proteomexchange.org) via the iProX partner repository^107^ with the dataset identifier PXD031467 under Project ID IPX0004027000. The WES data and transcriptomics data have been deposited in NODE (https://www.biosino.org/node) under the accession number OEP003433. The Mutation Annotation Format (MAF) file, mRNA expression matrix, the cohort-level tables for relative protein abundances and Phosphosite levels have been deposited in Figshare website (10.6084/m9.figshare.21340161).
