# Supplementary material for: Integrated proteogenomic characterization across major histological types of pituitary neuroendocrine tumors
Source: Cell Res. 2022 Oct 28;32(12):1047–67. doi: 10.1038/s41422-022-00736-5 (PMC9715725; doi:10.1038/s41422-022-00736-5)
Supplement: Supplementary file 1 — Fig. S1 [file 41422_2022_736_MOESM1_ESM.pdf]

a

TF lineage	Tumor name	Abbreviation	Hormone	Gene
PIT1	Lactotroph tumor	PRL	Prolactin (PRL)	PRL
	Somatotroph tumor	GH	Growth hormone (GH)	GH1
	Thyrotroph tumor	TSH	Thyroid stimulating hormone (TSH)	TSHB, CGA
	Silent PIT1 tumor	Silent PIT1	Non-functioning	-
TPIT	Corticotroph tumor	ACTH	Adrenocorticotropic Hormone (ACTH)	POMC
	Silent TPIT tumor	Silent TPIT	Non-functioning	-
SF1	Gonadotroph tumor	GN	Follicle Stimulating Hormone (FSH), and/or Luteinizing Hormone (LH)	FSHB, LHB, CGA
	Silent SF1 tumor	Silent SF1	Non-functioning	-
Null	Null-cell tumor	Null	Non-functioning	-
Pluri	Plurihormonal tumor	Pluri	Multiple hormones	-

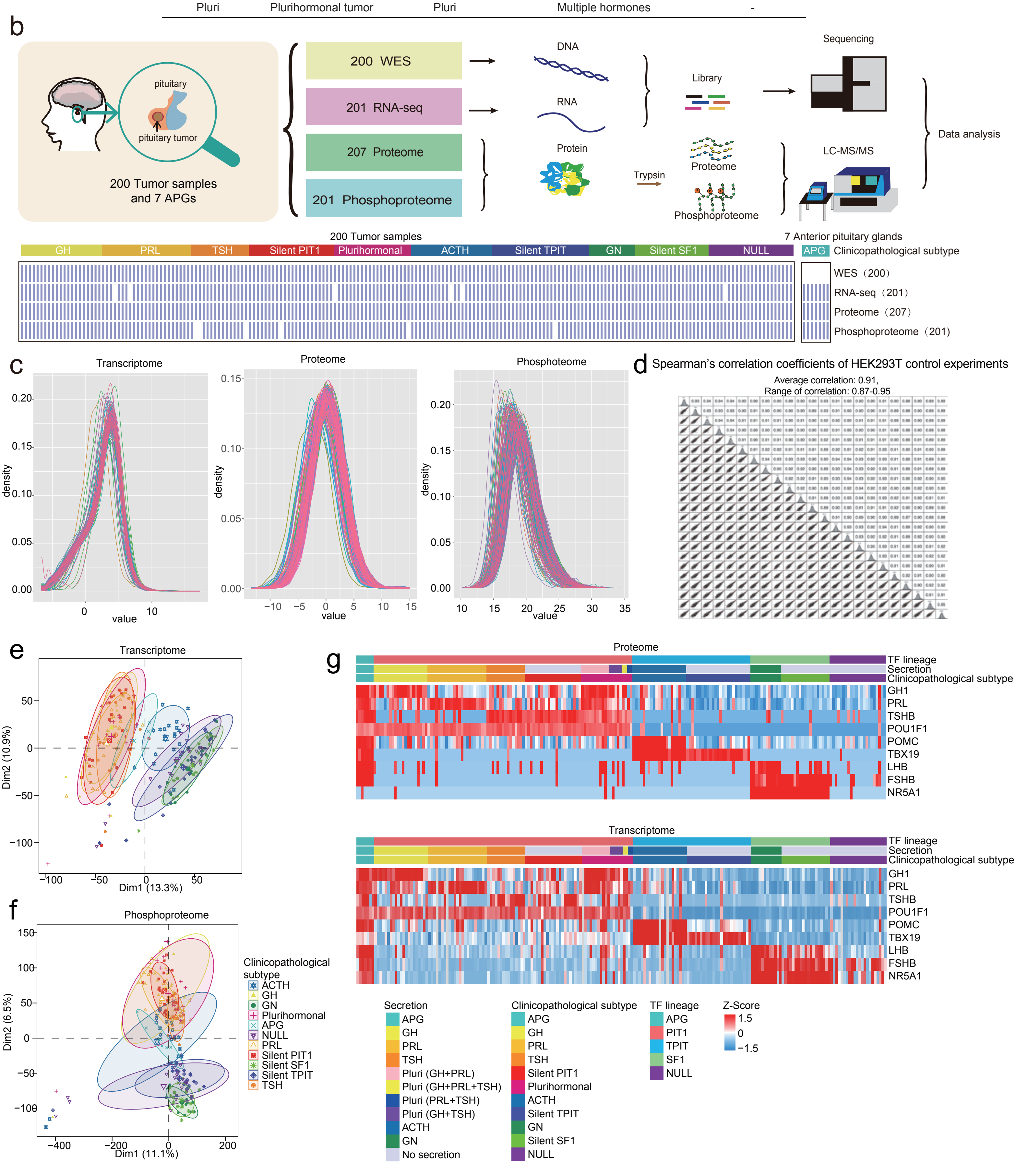

Supplementary information, Fig. S1 Proteogenomic landscape of PitNETs, related to Fig. 1.

a The clinicopathological classification (2017 WHO) of PitNETs.

b Schematic of the proteogenomic analyses of PitNETs (top). To facilitate homogeneity of samples, 200 fresh frozen tumors and donated 7 APGs were cryopulverized and aliquoted for genomic, transcriptomic, proteomic, and phosphoproteomic analyses (bottom).

c Distribution of mRNA, protein, phosphosite abundances in tumors by a density plot. A unimodal distribution (dip test) was observed. All of samples passed quality control.

d Longitudinal quality control of mass spectrometry using a tryptic digest of HEK293T cells. The top right panel presents the pairwise Spearman's correlation coefficients of the samples, and the bottom left panel depicts the pairwise comparison of 23 samples by scatterplots. An average correlation coefficient of 0.91 was observed.

e, f Principal component analysis of the transcriptomic and phosphoproteomic datasets based on clinicopathological subtype.

g Heatmaps showing the expression of known PitNET markers based on transcriptomics and proteomics data in each clinicopathological subtype.
