# Supplementary material for: Integrated proteogenomic characterization across major histological types of pituitary neuroendocrine tumors
Source: Cell Res. 2022 Oct 28;32(12):1047–67. doi: 10.1038/s41422-022-00736-5 (PMC9715725; doi:10.1038/s41422-022-00736-5)
Supplement: Supplementary file 2 — Fig. S2 [file 41422_2022_736_MOESM2_ESM.pdf]

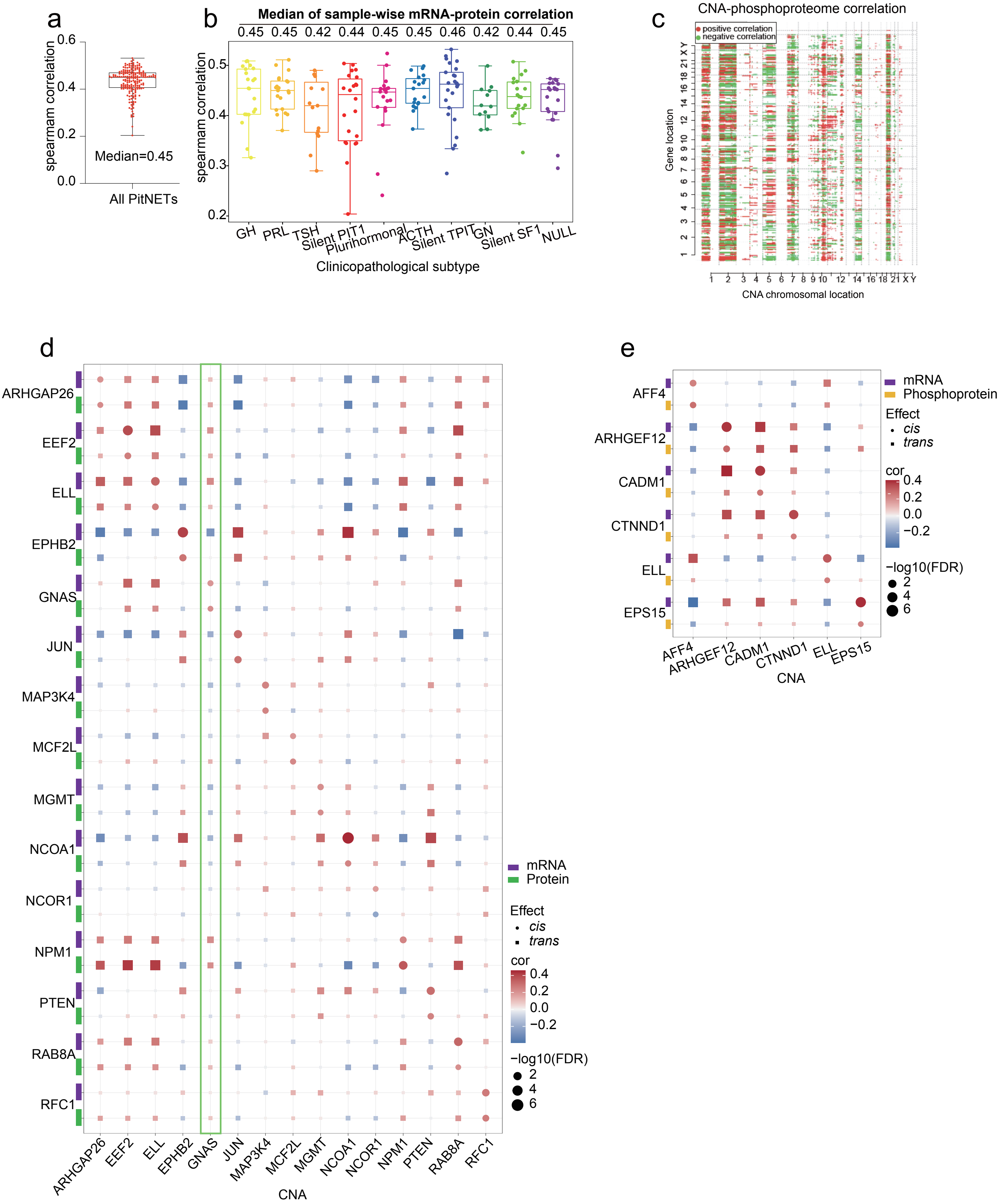

a
spearmam correlation
0.6
0.4
0.2
0.0
Median=0.45
All PitNETs
b
Median of sample-wise mRNA-protein correlation
0.45 0.45 0.42 0.44 0.45 0.45 0.46 0.42 0.44 0.45
spearmam correlation
0.5
0.4
0.3
0.2
GH
PRL
TSH
Silent PIT1
Plurihormonal
ACTH
Silent TPIT
GN
Silent SF1
NULL
Clinicopathological subtype
c
CNA-phosphoproteome correlation
positive correlation
negative correlation
Gene location
1 2 3 4 5 6 7 8 9 10 12 14 16 18 21 X Y
CNA chromosomal location
d
ARHGAP26
EEF2
ELL
EPHB2
GNAS
JUN
MAP3K4
MCF2L
MGMT
NCOA1
NCOR1
NPM1
PTEN
RAB8A
RFC1
mRNA
Protein
Effect
cis
trans
cor
0.4
0.2
0.0
−0.2
−log10(FDR)
2
4
6
ARHGAP26
EEF2
ELL
EPHB2
GNAS
JUN
MAP3K4
MCF2L
MGMT
NCOA1
NCOR1
NPM1
PTEN
RAB8A
RFC1
CNA
e
AFF4
ARHGEF12
CADM1
CTNND1
ELL
EPS15
mRNA
Phosphoprotein
Effect
cis
trans
cor
0.4
0.2
0.0
−0.2
−log10(FDR)
2
4
6
AFF4
ARHGEF12
CADM1
CTNND1
ELL
EPS15
CNA

Supplementary information, Fig. S2 Impact of CNAs on the transcriptome, proteome and phosphoproteome of PitNETs, related to Fig. 2.

a Boxplot showing sample-wise mRNA-protein correlations in all PitNETs.

b Median of sample-wise mRNA-protein correlations in each clinicopathological subtype.

c The panel shows the correlation of CNAs to phosphoprotein abundance, with significant positive correlations in red and negative correlations in green (Spearman's correlation, FDR < 0.05). Genes were sorted by chromosomal location on the *x* and *y* axes.

d Significant *cis* and *trans* effects of selected 15 CAGs on the expression of cancer associated mRNA and protein.

e Significant *cis* and *trans* effects of selected 6 CAGs on the expression of cancer associated mRNA and phosphoprotein.
