# Supplementary material for: Integrated proteogenomic characterization across major histological types of pituitary neuroendocrine tumors
Source: Cell Res. 2022 Oct 28;32(12):1047–67. doi: 10.1038/s41422-022-00736-5 (PMC9715725; doi:10.1038/s41422-022-00736-5)
Supplement: Supplementary file 3 — Fig. S3 [file 41422_2022_736_MOESM3_ESM.pdf]

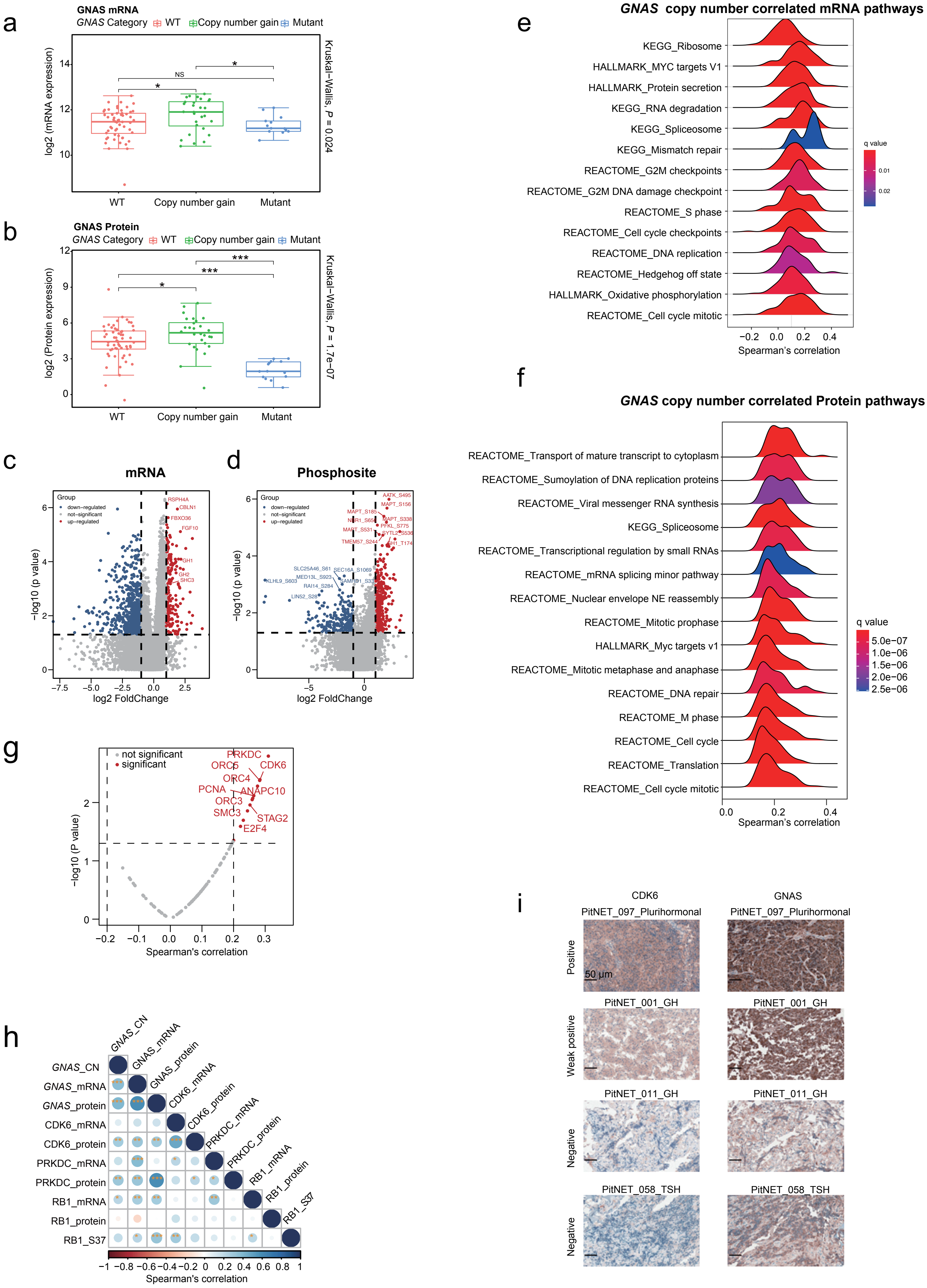

a
GNAS mRNA
GNAS Category WT Copy number gain Mutant
log2 (mRNA expression)
Kruskal−Wallis, P = 0.024
NS
WT
Copy number gain
Mutant
b
GNAS Protein
GNAS Category WT Copy number gain Mutant
log2 (Protein expression)
Kruskal−Wallis, P = 1.7e−07
WT
Copy number gain
Mutant
c
mRNA
Group
down−regulated
not−significant
up−regulated
RSPH4A
CBLN1
FBXO36
FGF10
−log10 (p value)
log2 FoldChange
d
Phosphosite
Group
down−regulated
not−significant
up−regulated
AATK_S495
MAPT_S156
MAPT_S185
MAPT_S338
PFKL_S775
MAPT_S531
TMEM57_S244
SLC25A46_S61
SEC16A_S1069
MED13L_S923
KLHL9_S603
RAI14_S284
LIN52_S28
−log10 (p value)
log2 FoldChange
e
GNAS copy number correlated mRNA pathways
KEGG_Ribosome
HALLMARK_MYC targets V1
HALLMARK_Protein secretion
KEGG_RNA degradation
KEGG_Spliceosome
KEGG_Mismatch repair
REACTOME_G2M checkpoints
REACTOME_G2M DNA damage checkpoint
REACTOME_S phase
REACTOME_Cell cycle checkpoints
REACTOME_DNA replication
REACTOME_Hedgehog off state
HALLMARK_Oxidative phosphorylation
REACTOME_Cell cycle mitotic
q value
0.01
0.02
Spearman's correlation
f
GNAS copy number correlated Protein pathways
REACTOME_Transport of mature transcript to cytoplasm
REACTOME_Sumoylation of DNA replication proteins
REACTOME_Viral messenger RNA synthesis
KEGG_Spliceosome
REACTOME_Transcriptional regulation by small RNAs
REACTOME_mRNA splicing minor pathway
REACTOME_Nuclear envelope NE reassembly
REACTOME_Mitotic prophase
HALLMARK_Myc targets v1
REACTOME_Mitotic metaphase and anaphase
REACTOME_DNA repair
REACTOME_M phase
REACTOME_Cell cycle
REACTOME_Translation
REACTOME_Cell cycle mitotic
q value
5.0e−07
1.0e−06
1.5e−06
2.0e−06
2.5e−06
Spearman's correlation
g
not significant
significant
PRKDC
ORC5
CDK6
ORC4
PCNA
ANAPC10
ORC3
SMC3
STAG2
E2F4
−log10 (P value)
Spearman's correlation
h
GNAS_CN
GNAS_mRNA
GNAS_protein
CDK6_mRNA
CDK6_protein
PRKDC_mRNA
PRKDC_protein
RB1_mRNA
RB1_protein
RB1_S37
Spearman's correlation
i
CDK6
GNAS
PitNET_097_Plurihormonal
Positive
50 μm
PitNET_001_GH
Weak positive
PitNET_011_GH
Negative
PitNET_058_TSH
Negative

Supplementary information, Fig. S3 Impact of *GNAS* mutation and *GNAS* copy number gain in PIT1 lineage PitNETs, related to Fig. 3.

a, b Boxplots showing the mRNA and protein abundance of *GNAS* among different *GNAS* altered categories. Kruskal-Wallis test was used to test if any of the differences among the subgroups were statistically significant. Wilcoxon rank-sum test was used to estimate the significance of two subgroups, $^{*}P < 0.05$, $^{***}P < 0.001$, NS (not significant).

c, d Volcano plots displaying the differentially expressed mRNAs and phosphosites in the *GNAS* mutant and *GNAS* wild-type patients after applying a two-fold change in expression with $P < 0.05$ (Wilcoxon rank-sum test). mRNAs and phosphosites that were significantly enriched in the *GNAS* mutant and *GNAS* wild-type patients were presented with red/blue-filled dots.

e, f Ridge plots showing the GSEA pathways enrichment based on the rank of *GNAS* copy number-mRNA (e)/protein (f) abundance correlations.

g Scatterplot showing the Spearman's correlation between *GNAS* copy number and cell cycle related proteins.

h Spearman's correlation of multi-omics abundance of genes in PIT1 lineage ($^{*}P < 0.05$, $^{**}P < 0.01$, $^{***}P < 0.001$).

i Representative pathology micrographs of IHC staining for CDK6 and GNAS. Scale bar is 50 μm.
