# Supplementary material for: Integrated proteogenomic characterization across major histological types of pituitary neuroendocrine tumors
Source: Cell Res. 2022 Oct 28;32(12):1047–67. doi: 10.1038/s41422-022-00736-5 (PMC9715725; doi:10.1038/s41422-022-00736-5)
Supplement: Supplementary file 4 — Fig. S4 [file 41422_2022_736_MOESM4_ESM.pdf]

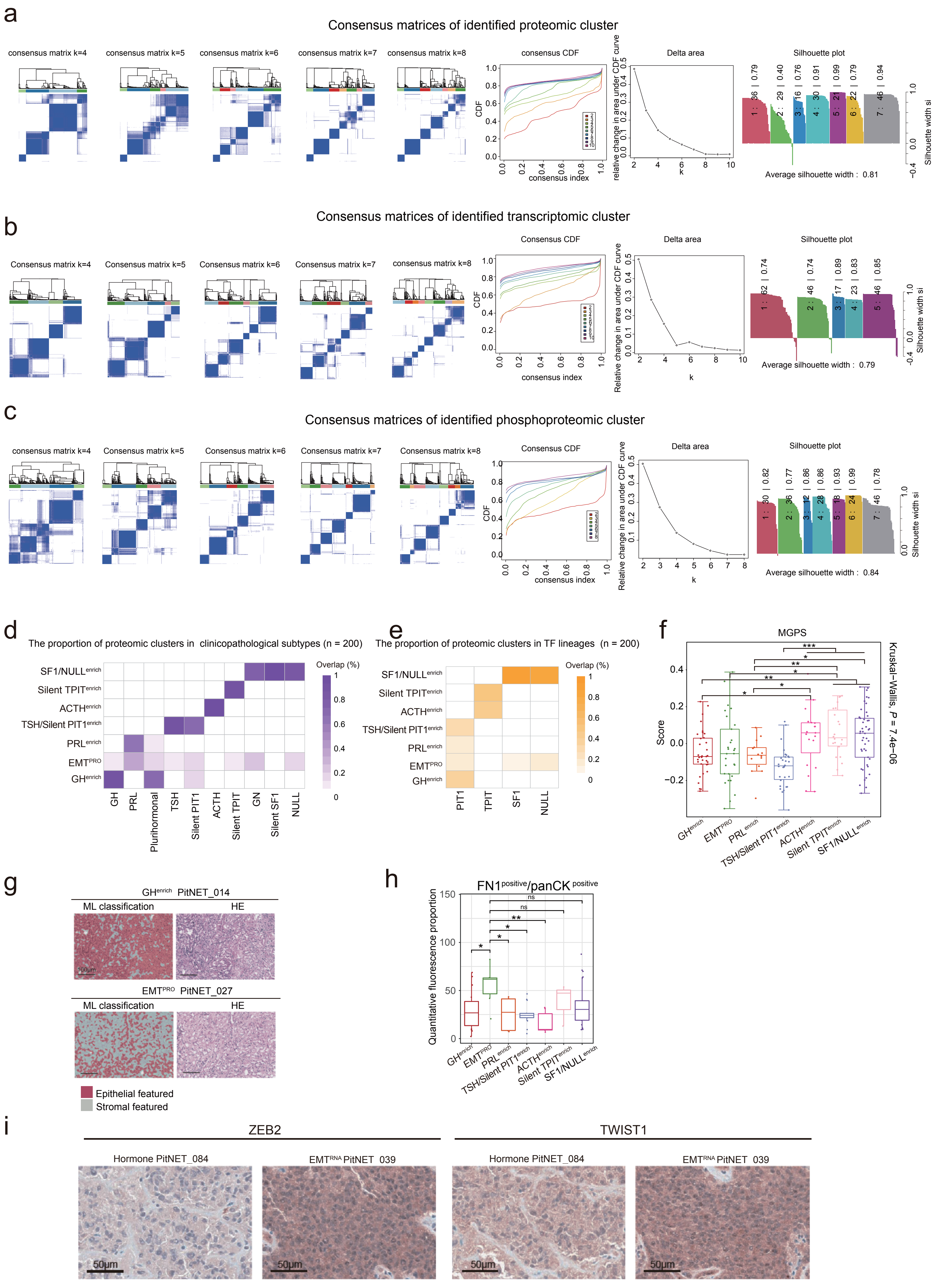
a
Consensus matrices of identified proteomic cluster
consensus matrix k=4
consensus matrix k=5
consensus matrix k=6
consensus matrix k=7
consensus matrix k=8
consensus CDF
Delta area
Silhouette plot
Average silhouette width : 0.81
b
Consensus matrices of identified transcriptomic cluster
Consensus matrix k=4
Consensus matrix k=5
Consensus matrix k=6
Consensus matrix k=7
consensus matrix k=8
Consensus CDF
Delta area
Silhouette plot
Average silhouette width : 0.79
c
Consensus matrices of identified phosphoproteomic cluster
consensus matrix k=4
Consensus matrix k=5
Consensus matrix k=6
Consensus matrix k=7
Consensus matrix k=8
Consensus CDF
Delta area
Silhouette plot
Average silhouette width : 0.84
d
The proportion of proteomic clusters in clinicopathological subtypes (n = 200)
e
The proportion of proteomic clusters in TF lineages (n = 200)
Overlap (%)
f
MGPS
Kruskal–Wallis, P = 7.4e−06
g
GHenrich PitNET_014
ML classification
HE
EMTPRO PitNET_027
Epithelial featured
Stromal featured
h
FN1positive/panCK positive
Quantitative fluorescence proportion
i
ZEB2
TWIST1
Hormone PitNET_084
EMTRNA PitNET_039
50μm

Supplementary information, Fig. S4 Consensus clustering for proteomic, transcriptomic and phosphoproteomic data, related to Fig. 4.

a-c Subgroups are identified based on proteomic (n = 200), transcriptomic (n = 194) and phosphoproteomic (n = 194) data by K-means consensus clustering upon their abundance. Consensus matrices, consensus cumulative distribution function (CDF) plot, delta area (change in CDF area) plot and silhouette plots are shown.

d, e The overlap of each proteomic cluster in clinicopathological subtypes (d) and TF lineages (e), respectively.

f Boxplots depicting the distribution of multi-gene proliferation scores (MGPS) inferred by ESTIMATE among tumors of the seven proteomic clusters. Wilcoxon rank-sum test was used to estimate the significance of two clusters (*$P < 0.05$, **$P < 0.01$, ***$P < 0.001$). Kruskal-Wallis test was used to test if any of the differences among the subgroups were statistically significant.

g The representative graphs of HE staining and machine learning (ML) classification in one GH^{enrich} and one EMT^{PRO}, respectively. Scale bar is 100 μm.

h Boxplot depicting the co-staining of panCK-positive and FN1-positive fluorescence areas among the seven proteomic clusters.

i IHC staining of representative pathology micrographs for ZEB2 and TWIST1. Scale bar is 50 μm.
