# Supplementary material for: Integrated proteogenomic characterization across major histological types of pituitary neuroendocrine tumors
Source: Cell Res. 2022 Oct 28;32(12):1047–67. doi: 10.1038/s41422-022-00736-5 (PMC9715725; doi:10.1038/s41422-022-00736-5)
Supplement: Supplementary file 5 — Fig. S5 [file 41422_2022_736_MOESM5_ESM.pdf]

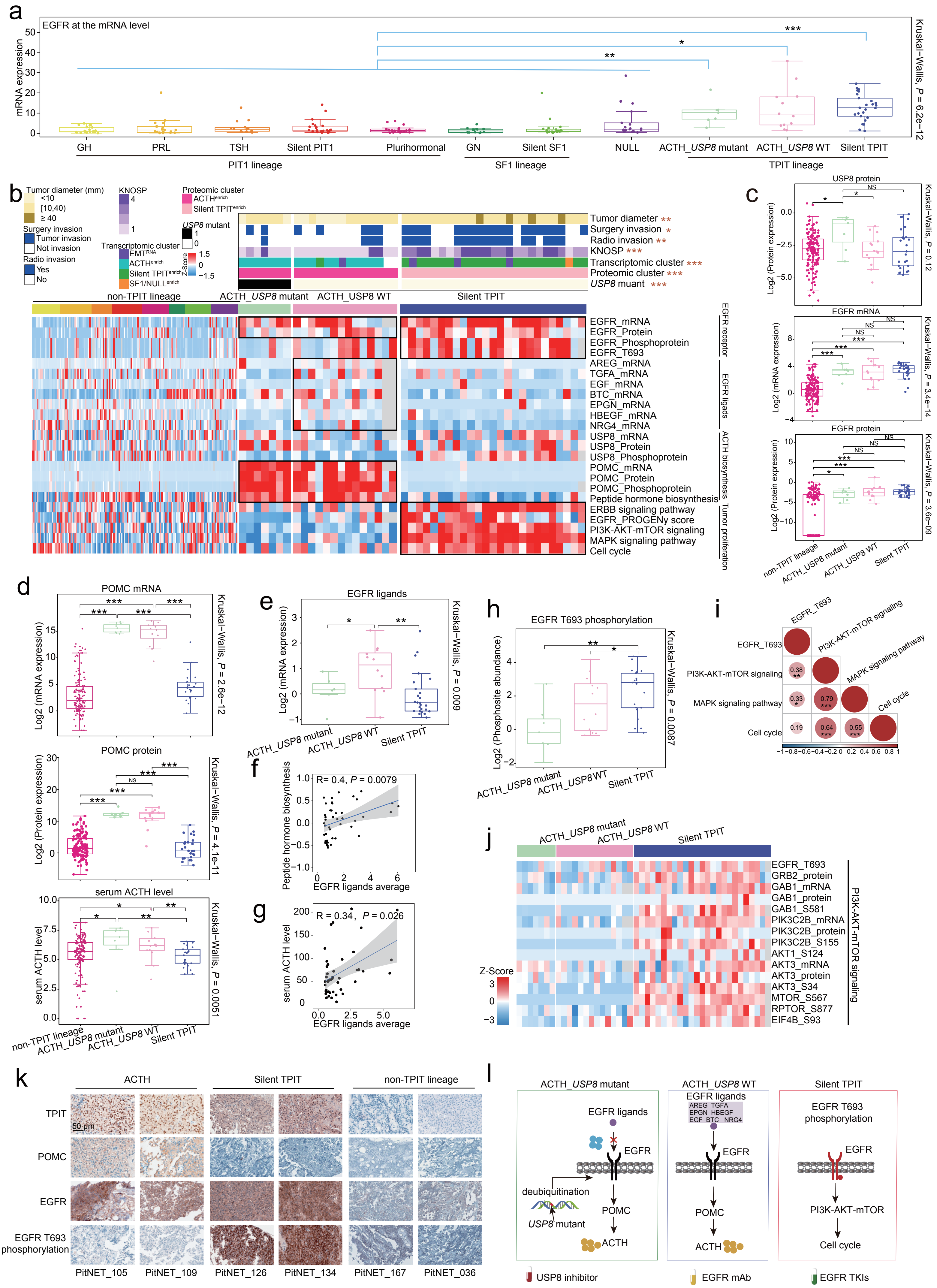

a
EGFR at the mRNA level
mRNA expression
Kruskal−Wallis, P = 6.2e−12
GH
PRL
TSH
Silent PIT1
Plurihormonal
GN
Silent SF1
NULL
ACTH_USP8 mutant
ACTH_USP8 WT
Silent TPIT
PIT1 lineage
SF1 lineage
TPIT lineage
b
Tumor diameter (mm)
<10
[10,40)
≥ 40
Surgery invasion
Tumor invasion
Not invasion
Radio invasion
Yes
No
KNOSP
Transcriptomic cluster
EMT^RNA
ACTH^enrich
Silent TPIT^enrich
SF1/NULL^enrich
Proteomic cluster
ACTH^enrich
Silent TPIT^enrich
USP8 mutant
Z-Score
Tumor diameter **
Surgery invasion *
Radio invasion **
KNOSP ***
Transcriptomic cluster ***
Proteomic cluster ***
USP8 muant ***
non-TPIT lineage
ACTH_USP8 mutant
ACTH_USP8 WT
Silent TPIT
EGFR_mRNA
EGFR_Protein
EGFR_Phosphoprotein
EGFR_T693
AREG_mRNA
TGFA_mRNA
EGF_mRNA
BTC_mRNA
EPGN_mRNA
HBEGF_mRNA
NRG4_mRNA
USP8_mRNA
USP8_Protein
USP8_Phosphoprotein
POMC_mRNA
POMC_Protein
POMC_Phosphoprotein
Peptide hormone biosynthesis
ERBB signaling pathway
EGFR_PROGENy score
PI3K-AKT-mTOR signaling
MAPK signaling pathway
Cell cycle
EGFR receptor
EGFR ligands
ACTH biosynthesis
Tumor proliferation
c
USP8 protein
Log2 (Protein expression)
Kruskal−Wallis, P = 0.12
EGFR mRNA
Log2 (mRNA expression)
Kruskal−Wallis, P = 3.4e−14
EGFR protein
Log2 (Protein expression)
Kruskal−Wallis, P = 3.6e−09
non-TPIT lineage
ACTH_USP8 mutant
ACTH_USP8 WT
Silent TPIT
d
POMC mRNA
Log2 (mRNA expression)
Kruskal−Wallis, P = 2.6e−12
POMC protein
Log2 (Protein expression)
Kruskal−Wallis, P = 4.1e−11
serum ACTH level
serum ACTH level
Kruskal−Wallis, P = 0.0051
non-TPIT lineage
ACTH_USP8 mutant
ACTH_USP8 WT
Silent TPIT
e
EGFR ligands
Log2 (mRNA expression)
Kruskal−Wallis, P = 0.009
ACTH_USP8 mutant
ACTH_USP8 WT
Silent TPIT
f
R= 0.4, P = 0.0079
Peptide hormone biosynthesis
EGFR ligands average
g
R = 0.34, P = 0.026
serum ACTH level
EGFR ligands average
h
EGFR T693 phosphorylation
Log2 (Phosphosite abundance)
Kruskal−Wallis, P = 0.0087
ACTH_USP8 mutant
ACTH_USP8 WT
Silent TPIT
i
EGFR_T693
PI3K-AKT-mTOR signaling
MAPK signaling pathway
Cell cycle
0.38
0.33
0.79
0.19
0.64
0.55
j
ACTH_USP8 mutant
ACTH_USP8 WT
Silent TPIT
Z-Score
EGFR_T693
GRB2_protein
GAB1_mRNA
GAB1_protein
GAB1_S581
PIK3C2B_mRNA
PIK3C2B_protein
PIK3C2B_S155
AKT1_S124
AKT3_mRNA
AKT3_protein
AKT3_S34
MTOR_S567
RPTOR_S877
EIF4B_S93
PI3K-AKT-mTOR signaling
k
ACTH
Silent TPIT
non-TPIT lineage
TPIT
POMC
EGFR
EGFR T693 phosphorylation
50 μm
PitNET_105
PitNET_109
PitNET_126
PitNET_134
PitNET_167
PitNET_036
l
ACTH_USP8 mutant
EGFR ligands
EGFR
deubiquitination
USP8 mutant
POMC
ACTH
ACTH_USP8 WT
EGFR ligands
AREG TGFA
EPGN HBEGF
EGF BTC NRG4
EGFR
POMC
ACTH
Silent TPIT
EGFR T693 phosphorylation
EGFR
PI3K-AKT-mTOR
Cell cycle
USP8 inhibitor
EGFR mAb
EGFR TKIs

Supplementary information, Fig. S5 Proteogenomic characterization of three EGFR activation modes in the TPIT lineage.

a The mRNA abundance of *EGFR* among ACTH_*USP8* mutant, ACTH_*USP8* WT, silent TPIT and other non-TPIT lineage tumors (Wilcoxon rank-sum test, $^*P < 0.05$, $^{**}P < 0.01$, $^{***}P < 0.001$). Kruskal-Wallis test was used to test whether any of the differences among the subgroups were statistically significant.

b Pathological and clinical annotations of 46 patients with TPIT lineage are provided (top heatmap) and Chi-square test was used for statistical tests ($^*P < 0.05$, $^{**}P < 0.01$, $^{***}P < 0.001$). EGFR receptor, EGFR ligands, and ACTH biosynthesis associated genes are presented at the mRNA, protein, and phosphorylation levels (medium heatmap). EGFR pathway activity was inferred using PROGENy based on mRNA data, and ssGSEA scores based on global proteomics data for biological pathways (bottom heatmap).

c Boxplots showing USP8 protein, *EGFR* mRNA, and EGFR protein abundance among the subgroups. Kruskal-Wallis test was used to test if any of the differences among the subgroups were statistically significant. Wilcoxon rank-sum test was used to estimate the significance of two subgroups, * P <0.05, $^{***}P < 0.001$, NS (not significant).

d Boxplots showing *POMC* mRNA, POMC protein and serum ACTH level among the subgroups. Kruskal-Wallis test was used to test if any of the differences among the subgroups were statistically significant. Wilcoxon rank-sum test was used to estimate the significance of two subgroups, $^*P < 0.05$, $^{**}P < 0.01$, $^{***}P < 0.001$, NS (not significant).

e Boxplot showing the average mRNA expression of EGFR ligands among the subtypes. Kruskal–Wallis test was used to test if any of the differences among the subgroups were statistically significant. Wilcoxon rank-sum test was used to estimate the significance of two subgroups, $^*P < 0.05$, $^{**}P < 0.01$.

f Scatterplot presenting the Spearman's correlation between EGFR ligands and peptide hormone biosynthesis.

g Scatterplot showing the Spearman's correlation between EGFR ligands average mRNA expression and serum ACTH level.

h Boxplot showing EGFR T693 phosphorylation levels among the three subgroups. Kruskal-Wallis test was used to test whether any of the differences among the subgroups were statistically significant. The Wilcoxon rank-sum test was used to estimate the significance of two subgroups, $^*P < 0.05$, $^{**}P < 0.01$.

i Spearman's correlation coefficients of EGFR T693 phosphorylation and EGFR downstream signaling pathways ($^*P < 0.05$, $^{**}P < 0.01$, $^{***}P < 0.001$).

j Heatmap comparing multi-omics profiles of PI3K-AKT-mTOR signaling pathway among ACTH_*USP8* mutant, ACTH_*USP8* WT, and silent TPIT groups.

k Representative micrographs of IHC staining for TPIT, POMC, EGFR, and EGFR T693 phosphorylated protein. Scale bar is 50 μm.

l Schematic diagram illustrating three modes of EGFR activation and potential treatments using USP8 inhibitor, EGFR mAb and EGFR TKIs in stratified TPIT lineage patients.
