# Supplementary material for: Integrated proteogenomic characterization across major histological types of pituitary neuroendocrine tumors
Source: Cell Res. 2022 Oct 28;32(12):1047–67. doi: 10.1038/s41422-022-00736-5 (PMC9715725; doi:10.1038/s41422-022-00736-5)
Supplement: Supplementary file 6 — Fig. S6 [file 41422_2022_736_MOESM6_ESM.pdf]

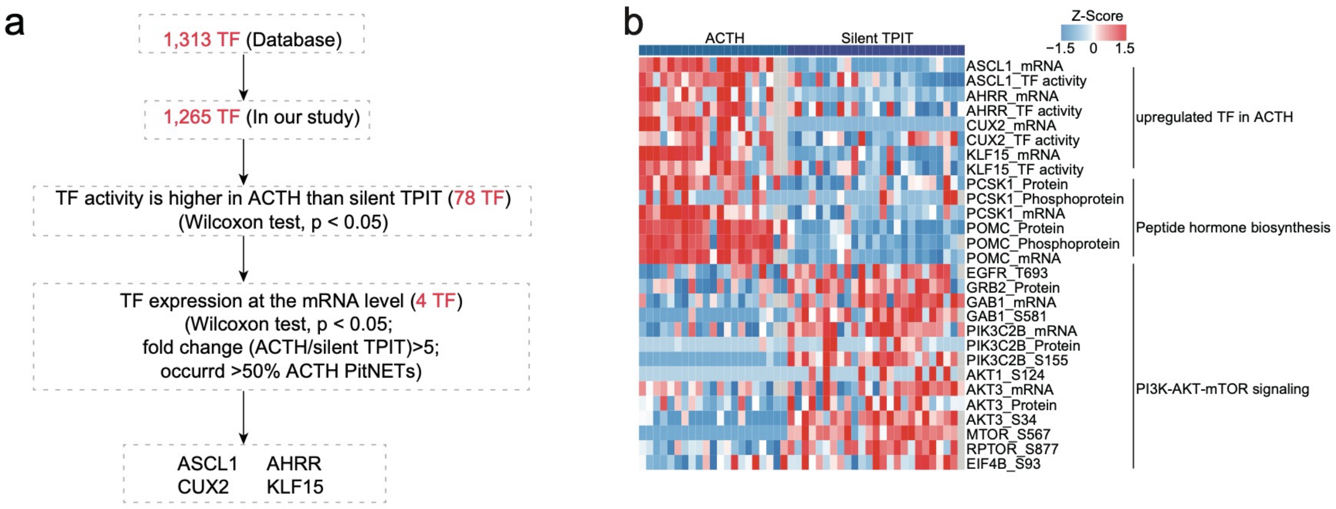

Supplementary information, Fig. S6 Different TFs between ACTH and silent TPIT PitNETs.

a Schematic of upregulated transcription factors screening in ACTH compared with silent TPIT PitNETs.

b Heatmap comparing multi-omics profiles of upregulated TFs, peptide hormone biosynthesis, and PI3K-AKT-mTOR signaling pathways between ACTH and silent TPIT PitNETs.
