# Supplementary material for: Integrated proteogenomic characterization across major histological types of pituitary neuroendocrine tumors
Source: Cell Res. 2022 Oct 28;32(12):1047–67. doi: 10.1038/s41422-022-00736-5 (PMC9715725; doi:10.1038/s41422-022-00736-5)
Supplement: Supplementary file 7 — Fig. S7 [file 41422_2022_736_MOESM7_ESM.pdf]

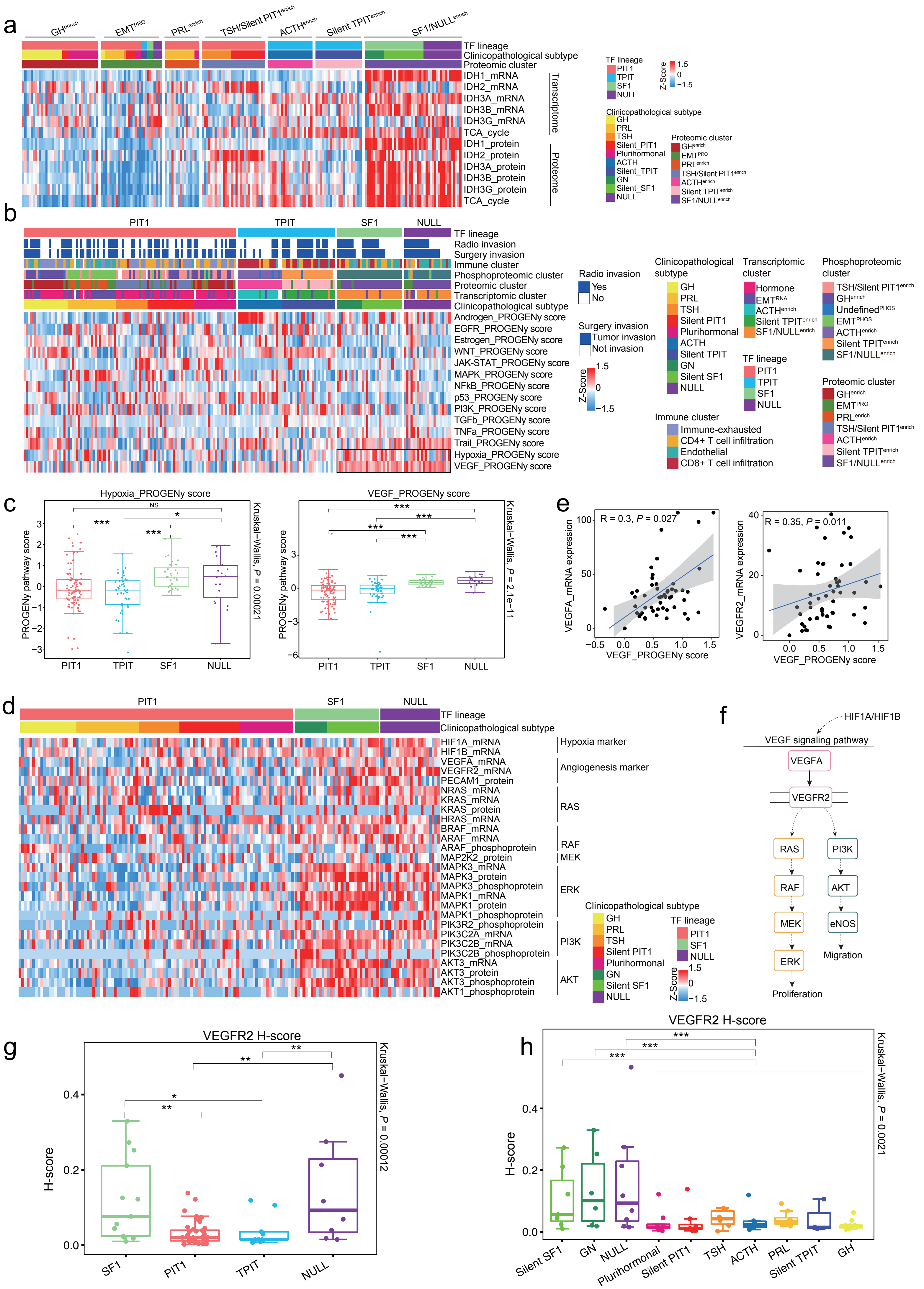
a
GH^enrich
EMT^PRO
PRL^enrich
TSH/Silent PIT1^enrich
ACTH^enrich
Silent TPIT^enrich
SF1/NULL^enrich
TF lineage
Clinicopathological subtype
Proteomic cluster
IDH1_mRNA
IDH2_mRNA
IDH3A_mRNA
IDH3B_mRNA
IDH3G_mRNA
TCA_cycle
IDH1_protein
IDH2_protein
IDH3A_protein
IDH3B_protein
IDH3G_protein
TCA_cycle
Transcriptome
Proteome
b
PIT1
TPIT
SF1
NULL
Radio invasion
Surgery invasion
Immune cluster
Phosphoproteomic cluster
Transcriptomic cluster
Androgen_PROGENy score
EGFR_PROGENy score
Estrogen_PROGENy score
WNT_PROGENy score
JAK-STAT_PROGENy score
MAPK_PROGENy score
NFkB_PROGENy score
p53_PROGENy score
PI3K_PROGENy score
TGFb_PROGENy score
TNFa_PROGENy score
Trail_PROGENy score
Hypoxia_PROGENy score
VEGF_PROGENy score
Yes
No
Tumor invasion
Not invasion
Z-Score
Hormone
EMT^RNA
Undefined^PHOS
EMT^PHOS
Immune cluster
Immune-exhausted
CD4+ T cell infiltration
Endothelial
CD8+ T cell infiltration
c
Hypoxia_PROGENy score
VEGF_PROGENy score
PROGENy pathway score
Kruskal–Wallis, P = 0.00021
Kruskal–Wallis, P = 2.1e–11
e
R = 0.3, P = 0.027
R = 0.35, P = 0.011
VEGFA_mRNA expression
VEGFR2_mRNA expression
VEGF_PROGENy score
d
HIF1A_mRNA
HIF1B_mRNA
VEGFA_mRNA
VEGFR2_mRNA
PECAM1_protein
NRAS_mRNA
KRAS_mRNA
KRAS_protein
HRAS_mRNA
BRAF_mRNA
ARAF_mRNA
ARAF_phosphoprotein
MAP2K2_protein
MAPK3_mRNA
MAPK3_protein
MAPK3_phosphoprotein
MAPK1_mRNA
MAPK1_protein
MAPK1_phosphoprotein
PIK3R2_phosphoprotein
PIK3C2A_mRNA
PIK3C2B_mRNA
PIK3C2B_phosphoprotein
AKT3_mRNA
AKT3_protein
AKT3_phosphoprotein
AKT1_phosphoprotein
Hypoxia marker
Angiogenesis marker
RAS
RAF
MEK
ERK
PI3K
AKT
f
HIF1A/HIF1B
VEGF signaling pathway
VEGFA
VEGFR2
RAS
RAF
MEK
ERK
Proliferation
PI3K
AKT
eNOS
Migration
g
VEGFR2 H-score
H-score
Kruskal–Wallis, P = 0.00012
h
VEGFR2 H-score
Kruskal–Wallis, P = 0.0021
Silent SF1
GN
NULL
Plurihormonal
Silent PIT1
TSH
ACTH
PRL
Silent TPIT
GH

Supplementary information, Fig. S7 Proteomic and transcriptomic alternations associated with tumorigenesis in SF1 lineage and NULL tumors.

a Heatmap analysis of mitochondrial citrate cycle enzymes and TCA cycle pathway in 200 PitNET cohort based on transcriptome and proteome data.

b Heatmap showing the PROGENy scores of 14 cancer-relevant pathways among PIT1, TPIT, SF1 lineages and NULL tumors.

c Boxplots showing the PROGENy scores of Hypoxia and VEGF signaling pathways. Kruskal-Wallis test was used to test if any of the differences among the subgroups were statistically significant. Wilcoxon rank-sum test was used to estimate the significance of two subgroups, $*P < 0.05$, $***P < 0.001$, NS (not significant).

d Heatmap showing the expression of hypoxia markers, angiogenesis markers, and other cancer-related genes at the mRNA, protein and phosphoprotein levels.

e Scatterplots describing Spearman's correlation of VEGF PROGENy score with the mRNA levels of VEGFA (left) and VEGFR2 (right).

f Summary model with activation of VEGF downstream pathways including RAS/RAF/MEK/ERK and PI3K-AKT which may lead to proliferation and migration in SF1 lineage and NULL tumors.

g Boxplot showing H-score of VEGFR2 among PIT1, TPIT, SF1 lineages and NULL tumors. Kruskal-Wallis test was used to test if any of the differences among the subgroups were statistically significant. Wilcoxon rank-sum test was used to estimate the significance of two subgroups, $*P < 0.05$, $**P < 0.01$.

h Boxplot showing H-score of VEGFR2 among silent SF1, GN, and NULL as compared with other clinicopathological subtypes (Wilcoxon rank-sum test, $***P < 0.001$). Kruskal-Wallis test was used to test if any of the differences among the subgroups were statistically significant.
