# Supplementary material for: Integrated proteogenomic characterization across major histological types of pituitary neuroendocrine tumors
Source: Cell Res. 2022 Oct 28;32(12):1047–67. doi: 10.1038/s41422-022-00736-5 (PMC9715725; doi:10.1038/s41422-022-00736-5)
Supplement: Supplementary file 8 — Fig. S8 [file 41422_2022_736_MOESM8_ESM.pdf]

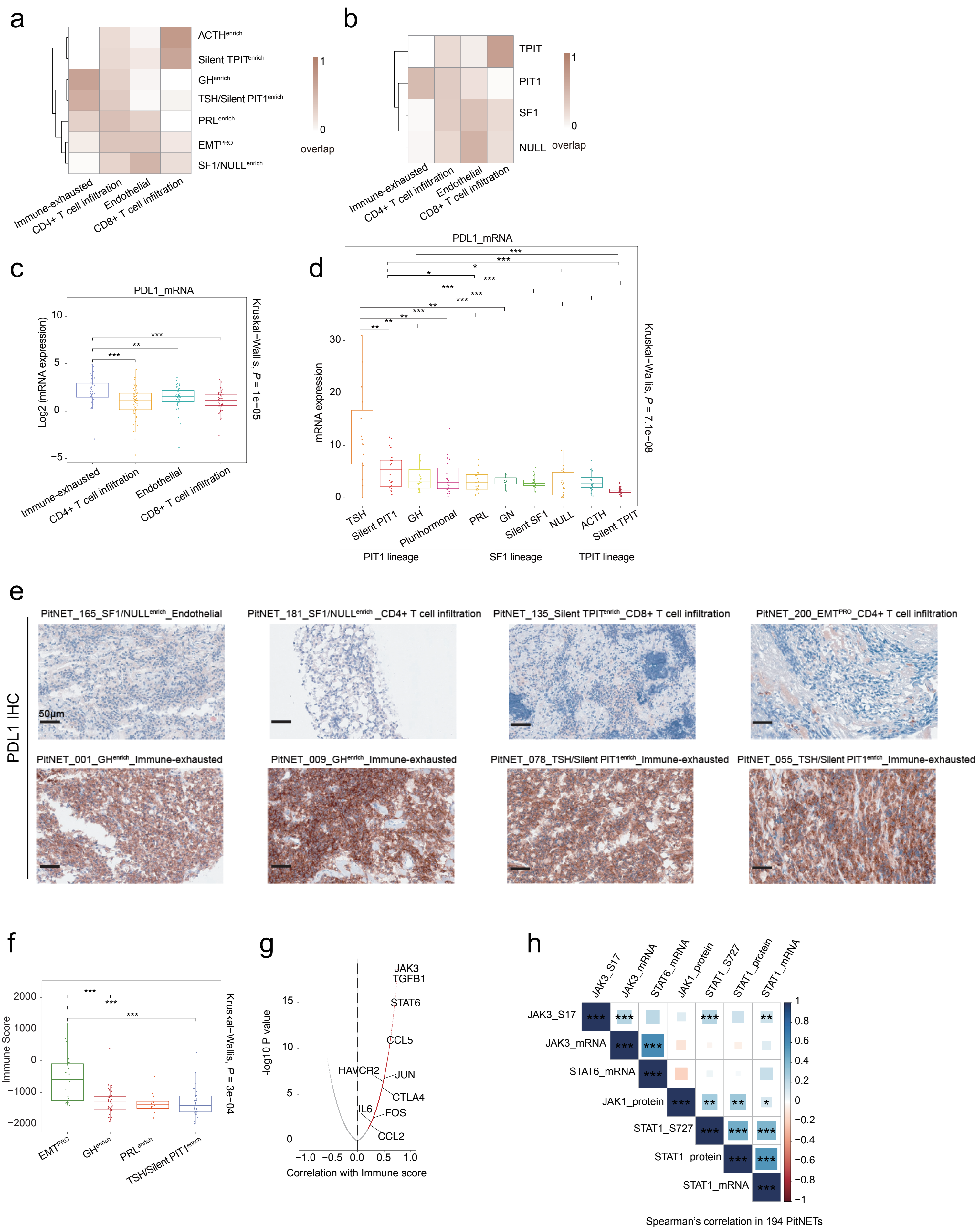

a
ACTHenrich
Silent TPITenrich
GHenrich
TSH/Silent PIT1enrich
PRLenrich
EMTPRO
SF1/NULLenrich
1
0
overlap
Immune-exhausted
CD4+ T cell infiltration
Endothelial
CD8+ T cell infiltration
b
TPIT
PIT1
SF1
NULL
1
0
overlap
Immune-exhausted
CD4+ T cell infiltration
Endothelial
CD8+ T cell infiltration
c
PDL1_mRNA
Log2 (mRNA expression)
Kruskal–Wallis, P = 1e−05
Immune-exhausted
CD4+ T cell infiltration
Endothelial
CD8+ T cell infiltration
d
PDL1_mRNA
mRNA expression
Kruskal–Wallis, P = 7.1e−08
TSH
Silent PIT1
GH
Plurihormonal
PRL
GN
Silent SF1
NULL
ACTH
Silent TPIT
PIT1 lineage
SF1 lineage
TPIT lineage
e
PDL1 IHC
PitNET_165_SF1/NULLenrich_Endothelial
PitNET_181_SF1/NULLenrich _CD4+ T cell infiltration
PitNET_135_Silent TPITenrich_CD8+ T cell infiltration
PitNET_200_EMTPRO_CD4+ T cell infiltration
50μm
PitNET_001_GHenrich_Immune-exhausted
PitNET_009_GHenrich_Immune-exhausted
PitNET_078_TSH/Silent PIT1enrich_Immune-exhausted
PitNET_055_TSH/Silent PIT1enrich_Immune-exhausted
f
Immune Score
Kruskal–Wallis, P = 3e−04
EMTPRO
GHenrich
PRL enrich
TSH/Silent PIT1enrich
g
-log10 P value
Correlation with Immune score
JAK3
TGFB1
STAT6
CCL5
HAVCR2
JUN
CTLA4
IL6
FOS
CCL2
h
JAK3_S17
JAK3_mRNA
STAT6_mRNA
JAK1_protein
STAT1_S727
STAT1_protein
STAT1_mRNA
Spearman's correlation in 194 PitNETs

Supplementary information, Fig. S8 Immune landscape in PitNETs, related to Fig. 5.

a Heatmap showing the comparison between immune clusters (columns) with proteomic clusters (rows). Each row sums to one, with different entries showing the proportion of tumors allocated to different immune clusters.

b Heatmap showing the comparison between immune clusters (columns) with TF lineages (rows). Each row sums to one, with different entries showing the proportion of tumors allocated to different immune clusters.

c Boxplot showing the mRNA expression of *PDL1* among the four immune clusters. Kruskal-Wallis test was used to test if any of the differences among the subgroups were statistically significant. Wilcoxon rank-sum test was used to estimate the significance of two subgroups, $^{**}P < 0.01$, $^{***}P < 0.001$.

d Boxplot showing the mRNA expression of PDL1 among the ten clinicopathological subtypes. Kruskal-Wallis test was used to test if any of the differences among the subgroups were statistically significant. Wilcoxon rank-sum test was used to estimate the significance of two subgroups, $^{**}P < 0.01$, $^{***}P < 0.001$.

e Representative graphs of PDL1 staining in SF1/NULL^{enrich}, silent TPIT^{enrich}, EMT^{PRO}, GH^{enrich}, and TSH/silent PIT1^{enrich} clusters. Scale bar is 50 μm.

f Boxplot showing the immune score among four proteomic clusters within PIT1 lineage. Kruskal-Wallis test was used to test if any of the differences among the subgroups were statistically significant. Wilcoxon rank-sum test was used to estimate the significance of two subgroups, $^{***}P < 0.001$.

g Volcano plot showing the Spearman's correlation (*x*-axis) versus *P* value (*y*-axis) between immune score and genes at the mRNA level.

h Spearman's correlation among JAK1, JAK3, STAT1 and STAT6 at the mRNA, protein and phosphorylation levels in 194 PitNETs ($^{*}P < 0.05$, $^{**}P < 0.01$, $^{***}P < 0.001$).
