# Supplementary material for: Integrated proteogenomic characterization across major histological types of pituitary neuroendocrine tumors
Source: Cell Res. 2022 Oct 28;32(12):1047–67. doi: 10.1038/s41422-022-00736-5 (PMC9715725; doi:10.1038/s41422-022-00736-5)
Supplement: Supplementary file 9 — Fig. S9 [file 41422_2022_736_MOESM9_ESM.pdf]

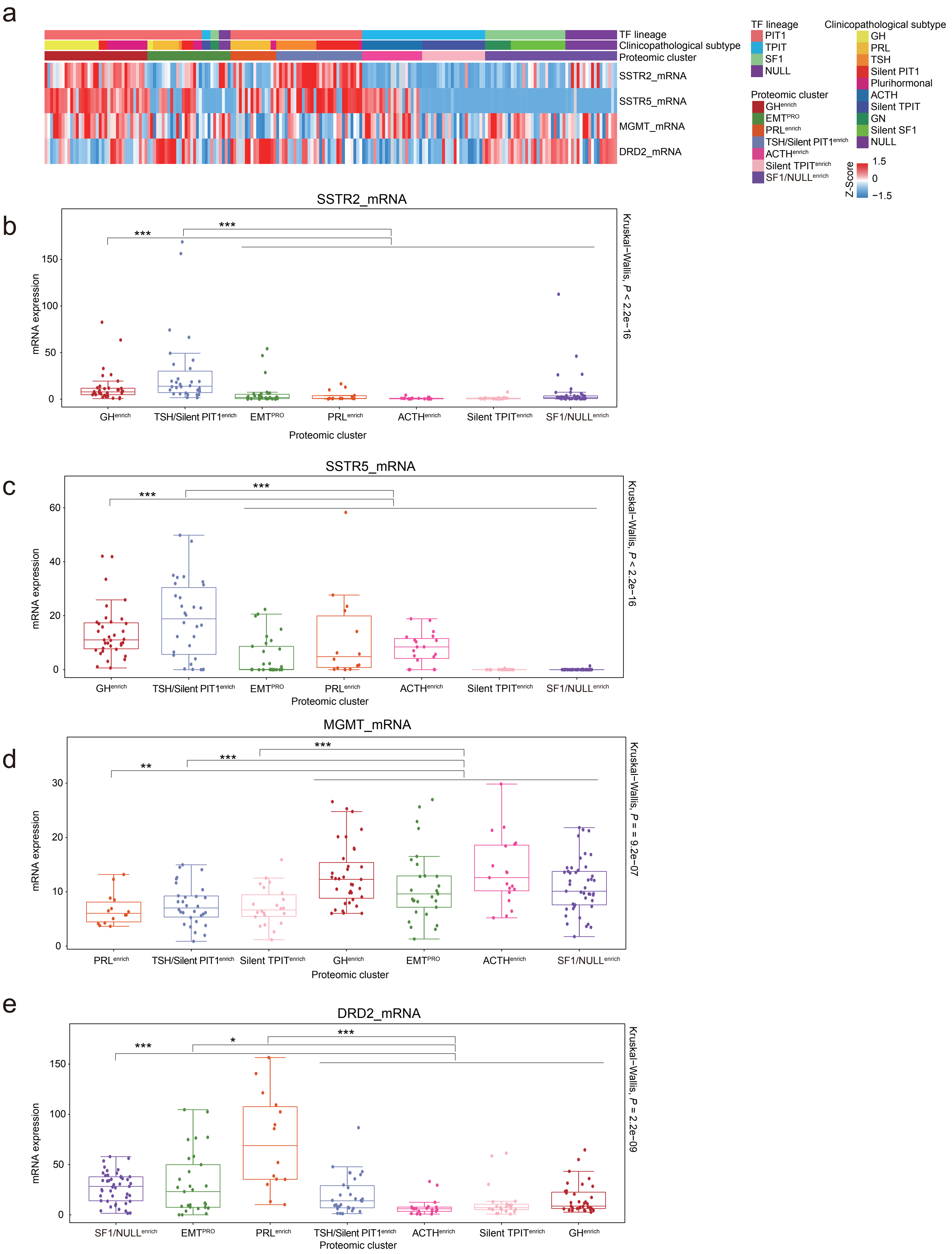
a
TF lineage
Clinicopathological subtype
Proteomic cluster
SSTR2_mRNA
SSTR5_mRNA
MGMT_mRNA
DRD2_mRNA
TF lineage
PIT1
TPIT
SF1
NULL
Proteomic cluster
GHenrich
EMTPRO
PRLenrich
TSH/Silent PIT1enrich
ACTHenrich
Silent TPITenrich
SF1/NULLenrich
Clinicopathological subtype
GH
PRL
TSH
Silent PIT1
Plurihormonal
ACTH
Silent TPIT
GN
Silent SF1
NULL
Z-Score
1.5
0
−1.5
b
SSTR2_mRNA
mRNA expression
Proteomic cluster
Kruskal–Wallis, P < 2.2e−16
GHenrich
TSH/Silent PIT1enrich
EMTPRO
PRLenrich
ACTHenrich
Silent TPITenrich
SF1/NULLenrich
c
SSTR5_mRNA
mRNA expression
Proteomic cluster
Kruskal–Wallis, P < 2.2e−16
GHenrich
TSH/Silent PIT1enrich
EMTPRO
PRLenrich
ACTHenrich
Silent TPITenrich
SF1/NULLenrich
d
MGMT_mRNA
mRNA expression
Proteomic cluster
Kruskal–Wallis, P = 9.2e−07
PRLenrich
TSH/Silent PIT1enrich
Silent TPITenrich
GHenrich
EMTPRO
ACTHenrich
SF1/NULLenrich
e
DRD2_mRNA
mRNA expression
Proteomic cluster
Kruskal–Wallis, P = 2.2e−09
SF1/NULLenrich
EMTPRO
PRLenrich
TSH/Silent PIT1enrich
ACTHenrich
Silent TPITenrich
GHenrich

Supplementary information, Fig. S9 The mRNA expression levels of targets of FDA-approved drugs in all 200 PitNETs, related to Fig. 6.

a Heatmap comparing the mRNA expression of *SSTR2*, *SSTR5*, *MGMT* and *DRD2* among the seven proteomic clusters.

b, c Boxplot showing mRNA expression of *SSTR2* **(b)** and *SSTR5* **(c)** was significantly elevated in GH^{enrich} and TSH/silent $PIT1^{enrich}$ than in other proteomic clusters (Wilcoxon rank-sum test, ***$P < 0.001$). Kruskal-Wallis test was used to test if any of the differences among the subgroups were statistically significant.

d Boxplot showing mRNA expression of *MGMT* was significantly lower in PRL^{enrich}, TSH/silent $PIT1^{enrich}$, and silent $TPIT^{enrich}$ than in other proteomic clusters, respectively (Wilcoxon rank-sum test, **$P < 0.001$, ***$P < 0.001$). Kruskal-Wallis test was used to test if any of the differences among the subgroups were statistically significant.

e Boxplot showing mRNA expression of *DRD2* was significantly elevated in SF1/$NULL^{enrich}$, EMT^{PRO}, and PRL^{enrich} than in other proteomic clusters, respectively (Wilcoxon rank-sum test, *$P < 0.05$, ***$P < 0.001$). Kruskal-Wallis test was used to test if any of the differences among the subgroups were statistically significant.
